# Phospho-RPA2 predicts response to platinum and PARP inhibitors in homologous recombination–proficient ovarian cancer

**DOI:** 10.1172/JCI189511

**Published:** 2025-05-20

**Authors:** Angela Schab, Amanda Compadre, Rebecca Drexler, Megan Loeb, Kevin Rodriguez, Joshua Brill, Shariska Harrington, Carmen Sandoval, Brooke Sanders, Lindsay Kuroki, Carolyn McCourt, Andrea R. Hagemann, Premal Thaker, David Mutch, Matthew Powell, Violeta Serra, Ian S. Hagemann, Ann E. Walts, Beth Y. Karlan, Sandra Orsulic, Katherine C. Fuh, Lulu Sun, Priyanka Verma, Elena Lomonosova, Peinan Zhao, Dineo Khabele, Mary M. Mullen

**Affiliations:** 1 Division of Gynecologic Oncology, Department of Obstetrics and Gynecology, Washington University, St. Louis, Missouri, USA.; 2 Center for Reproductive Health Sciences, Department of Obstetrics and Gynecology, and; 3 Department of Obstetrics and Gynecology, Mayo Clinic, Rochester, Minnesota, USA.; 4 Experimental Therapeutics Group, Vall d’Hebron Institute of Oncology, Barcelona, Spain.; 5 Department of Pathology and Immunology, Washington University School of Medicine, St. Louis, Missouri, USA.; 6 Department of Pathology and Laboratory Medicine, Cedars-Sinai Medical Center, Los Angeles, California, USA.; 7 Division of Gynecologic Oncology, Department of Obstetrics and Gynecology, David Geffen School of Medicine, University of California, Los Angeles, Los Angeles, California, USA.; 8 Veterans Affairs, Greater Los Angeles Healthcare System, Los Angeles, California, USA.; 9 Division of Gynecologic Oncology, Department of Obstetrics and Gynecology, University of California, San Francisco, San Francisco, California, USA.; 10 Division of Oncology, Department of Medicine, Washington University School of Medicine, St. Louis, Missouri, USA.

**Keywords:** Clinical Research, Oncology, DNA repair, Molecular diagnosis, Obstetrics/gynecology

## Abstract

**BACKGROUND:**

Treatment of tubo-ovarian high-grade serous carcinoma (HGSC) includes cytoreductive surgery, platinum-based chemotherapy, and often poly(ADP-ribose) polymerase (PARP) inhibitors. While homologous recombination (HR) deficiency is a well-established predictor of therapy sensitivity, over 50% of HR-proficient HGSCs also exhibit sensitivity. Currently, there are no biomarkers to identify which HR-proficient HGSCs will be sensitive to standard-of-care therapy. Replication stress may serve as a key determinant of response.

**METHODS:**

We evaluated phospho–RPA2-T21 (p-RPA2) foci via immunofluorescence as a biomarker of replication stress in formalin-fixed, paraffin-embedded HGSC samples collected at diagnosis from patients treated with platinum chemotherapy (discovery cohort, *n* = 31; validation cohort, *n* = 244) or PARP inhibitors (*n* = 63). Recurrent HGSCs (*n* = 38) were also analyzed. p-RPA2 score was calculated using automated imaging analysis.

**RESULTS:**

Samples were defined as p-RPA2-high if more than 16% of cells had ≥2 p-RPA2 foci on automated analysis. In the discovery cohort, HR-proficient, p-RPA2-high HGSCs demonstrated significantly higher rates of a chemotherapy response score of 3 to platinum chemotherapy than HR-proficient, p-RPA2-low HGSCs. In the validation cohort, patients with HR-proficient, p-RPA2-high HGSCs had significantly longer survival after platinum treatment than those with HR-proficient, p-RPA2-low HGSCs. Additionally, the p-RPA2 assay effectively predicted survival outcomes in patients treated with PARP inhibitors and in recurrent HGSC samples.

**CONCLUSION:**

Our study underscores the importance of considering replication stress markers, such as p-RPA2, alongside HR status in therapeutic planning. This approach has the potential to increase the number of patients receiving effective therapy while reducing unnecessary toxicity.

**FUNDING:**

The Reproductive Scientist Development Program, GOG Foundation, Pilot Translational and Clinical Studies function of the Washington University Institute of Clinical and Translational Sciences, the Foundation for Barnes-Jewish Hospital, Washington University School of Medicine Dean’s Scholar Program, The Cancer Biology Pathway Training Grant (5T32CA113275-17), The Lucy, Anarcha, and Betsey (L.A.B.) Award from the Department of Obstetrics and Gynecology at Washington University School of Medicine, and Veterans Affairs Office of Research and Development (I01BX006020).

## Abstract 2

The replication stress marker, phospho-RPA2, predicts response to platinum chemotherapy and PARP inhibitors in homologous recombination-proficient ovarian cancer, offering a biomarker to improve patient outcomes.

## Graphical abstract

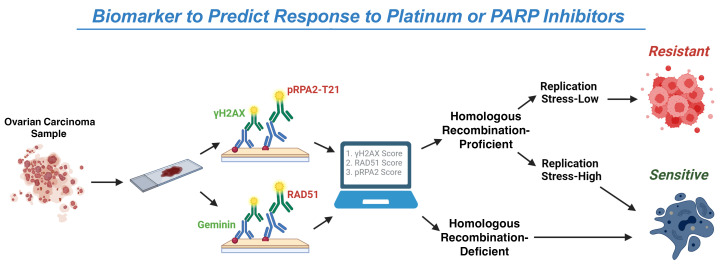

## Introduction

Ovarian cancer is treated with a combination of cytoreductive surgery and platinum-based chemotherapy (1–4). Additionally, current guidelines offer poly(ADP-ribose) polymerase (PARP) inhibitors, such as olaparib or niraparib, as maintenance therapy for all patients who are PARP inhibitor naive and have had a partial or complete response to platinum chemotherapy (5–7). While these therapies are effective initially, over 85% of patients with advanced ovarian cancer eventually develop resistance to standard-of-care platinum chemotherapy and PARP inhibitors, and most die within 5 years of diagnosis (1–4). A major determinant of cancer sensitivity or resistance to these therapies is whether cancer cells can perform homologous recombination (HR) to repair double-stranded DNA (dsDNA) breaks created by platinum-induced DNA adducts or PARP inhibitors (8). Previously, we developed an automated immunofluorescence assay to evaluate functional HR in formalin-fixed, paraffin-embedded (FFPE) tubo-ovarian high-grade serous carcinomas (HGSCs) (9). Our assay measures foci of RAD51, as binding of this protein to DNA relies on many upstream HR events and is a functional readout for HR proficiency (10, 11). We and others found that while most RAD51-low or functionally HR-deficient HGSCs respond to therapy, over 50% of RAD51-high or HR-proficient HGSCs are also sensitive to these therapies. This suggests that HR-independent mechanisms play a substantial role in determining therapeutic response (9, 12, 13). These findings are supported by clinical trial data demonstrating that over 65% of women with BRCA-mutated or HR-deficient HGSCs achieve a survival rate exceeding 90 months when treated with platinum chemotherapy followed by PARP inhibitors (9, 12–14). Similarly, there is a subset of up to 30% of women with HR-proficient HGSCs that experiences survival extending beyond 70 months after platinum and PARP inhibitor treatment (15, 16). This is in stark contrast to the median survival of women with HR-proficient HGSCs after platinum and PARP inhibitor treatment of 36.6 months (16). Together, these data indicate the need for a more accurate assay to determine whether patients with HR-proficient HGSCs will benefit from platinum chemotherapy and PARP inhibitors. Such an assay could improve treatment decisions, expand the number of patients receiving effective therapy, and reduce unnecessary toxicity (17–22).

An important determinant of platinum and PARP inhibitor sensitivity in HR-proficient cancer cells in vitro is replication stress (23, 24). Replication stress occurs when, in response to genotoxic damage, replication forks slow and initiate DNA damage tolerance pathways, leading to the accumulation of single-stranded DNA (25, 26). If the cell repairs the damage, then the replication stress resolves. However, if replication stress is excessive, the replication fork collapses, resulting in multiple double-stranded DNA breaks and cell death even in HR-proficient cells. Specifically, replication stress events such as fork degradation and single-stranded DNA (ssDNA) gap formation are directly linked to treatment sensitivity independent of HR status (23, 24, 27–29). A key protein involved in replication stress is replication protein A (RPA). RPA, which is composed of 3 subunits, binds to the ssDNA that accumulates in response to stalled replication forks. Next, one of the 3 subunits, RPA2, is phosphorylated sequentially at multiple sites. Thr21 is the last site to be phosphorylated and indicates impending replication catastrophe and irreparable damage (30–32). Therefore, we hypothesized that HR-proficient HGSC cells with high Thr21 phospho-RPA2 (p-RPA2) would have a better response to platinum chemotherapy or PARP inhibitors than RAD51-high HGSCs with low p-RPA2.

Here, we show that p-RPA2 foci can be reliably identified in FFPE HGSC samples. Additionally, using samples from multiple cohorts of patients with ovarian cancer, we show that HR-proficient, p-RPA2-high HGSCs had significantly better responses to platinum chemotherapy and PARP inhibitors than HR-proficient, p-RPA2-low HGSCs. We also developed automated quantification methods to objectively measure functional HR status and p-RPA2 foci, which should enable testing of this new assay in clinical trials.

## Results

### p-RPA2 foci reflect replication stress and can be quantified in patient-derived ovarian cancer samples.

We hypothesized that HR-proficient HGSC cells with high Thr21-phosphorylated RPA2 (p-RPA2) foci would respond better to platinum chemotherapy than those with low p-RPA2 foci. To begin to explore this, we stained 9 HR-proficient primary ovarian cancer cells (POVs) (Supplemental Figure 1A; supplemental material available online with this article; https://doi.org/10.1172/JCI189511DS1) with an antibody against p-RPA2. Clinical genomic analysis confirmed *BRCA1/2* wild-type status in 7 POVs, with one *BRCA2*-mutant case and one lacking sequencing data. RAD51 foci assay in these 2 samples showed intact HR repair (33), including the *BRCA2*-mutant case. We then counted the number of p-RPA2 foci at baseline per cell and calculated in vitro carboplatin sensitivity (IC_50_) for each POV. Platinum-resistant samples (*n* = 5) had higher IC_50_ values than platinum-sensitive ones (*n* = 3) (31.2 vs. 15.9 μM, *P* = 0.2), suggesting a trend toward increased resistance. While not statistically significant owing to the limited sample size, the pattern reflects expected clinical behavior. The number of p-RPA2 foci significantly negatively correlated with carboplatin IC_50_ (Spearman’s *r* = –0.82, *P* = 0.01) (Supplemental Figure 1B). These data suggested that p-RPA2 is a useful biomarker for carboplatin sensitivity in patient-derived ovarian cancer cells.

Non-fixed primary cells are not readily available in the clinic. Therefore, to facilitate the translation of our findings, we next sought to determine the accuracy of p-RPA2 as a biomarker for replication stress in FFPE samples. To do so, we treated 2 HR-proficient high-grade serous ovarian cancer cell lines (TYKNU and OVCAR8) with increasing doses of hydroxyurea to induce replication stress. We then formalin-fixed and paraffin-embedded the lines as cell blocks and evaluated p-RPA2 foci by immunofluorescence (9). Each sample was assigned a p-RPA2 score, defined as the percentage of cells with at least 5 p-RPA2 foci. With increasing doses of hydroxyurea, we observed an increase in p-RPA2 score (Supplemental Figure 2, A and B). To validate the specificity of p–RPA2-Thr21 antibody, we used siRNA to knock down RPA2 in the same cell lines, created FFPE cell blocks, and evaluated p-RPA2 foci. We observed significantly lower p-RPA2 scores in cells in which RPA2 was knocked down, both with and without hydroxyurea treatment (Supplemental Figure 2, C and D). We concluded that the p–RPA2-Thr21 antibody is sensitive and specific, that p-RPA2 foci staining accurately reflects replication stress, and that the assay can be conducted on FFPE HGSC samples.

### p-RPA2 foci in FFPE patient samples predict response to platinum chemotherapy and survival outcomes in RAD51-high HGSCs.

To investigate the accuracy of p-RPA2 score in predicting response to platinum chemotherapy, we evaluated the association between p-RPA2 foci and platinum chemotherapy response in a discovery cohort of FFPE biopsies obtained from primary HGSC sites before patients received neoadjuvant carboplatin (*n* = 31) (Table 1). All patients had advanced-stage disease and at least a component of HGSC histology. All samples were screened by a board-certified pathologist for diagnosis, cellularity, and necrosis. Samples had previously been scored as RAD51-high (HR-proficient) or RAD51-low (HR-deficient) using RAD51 foci as described (9).

**Figure 1:**
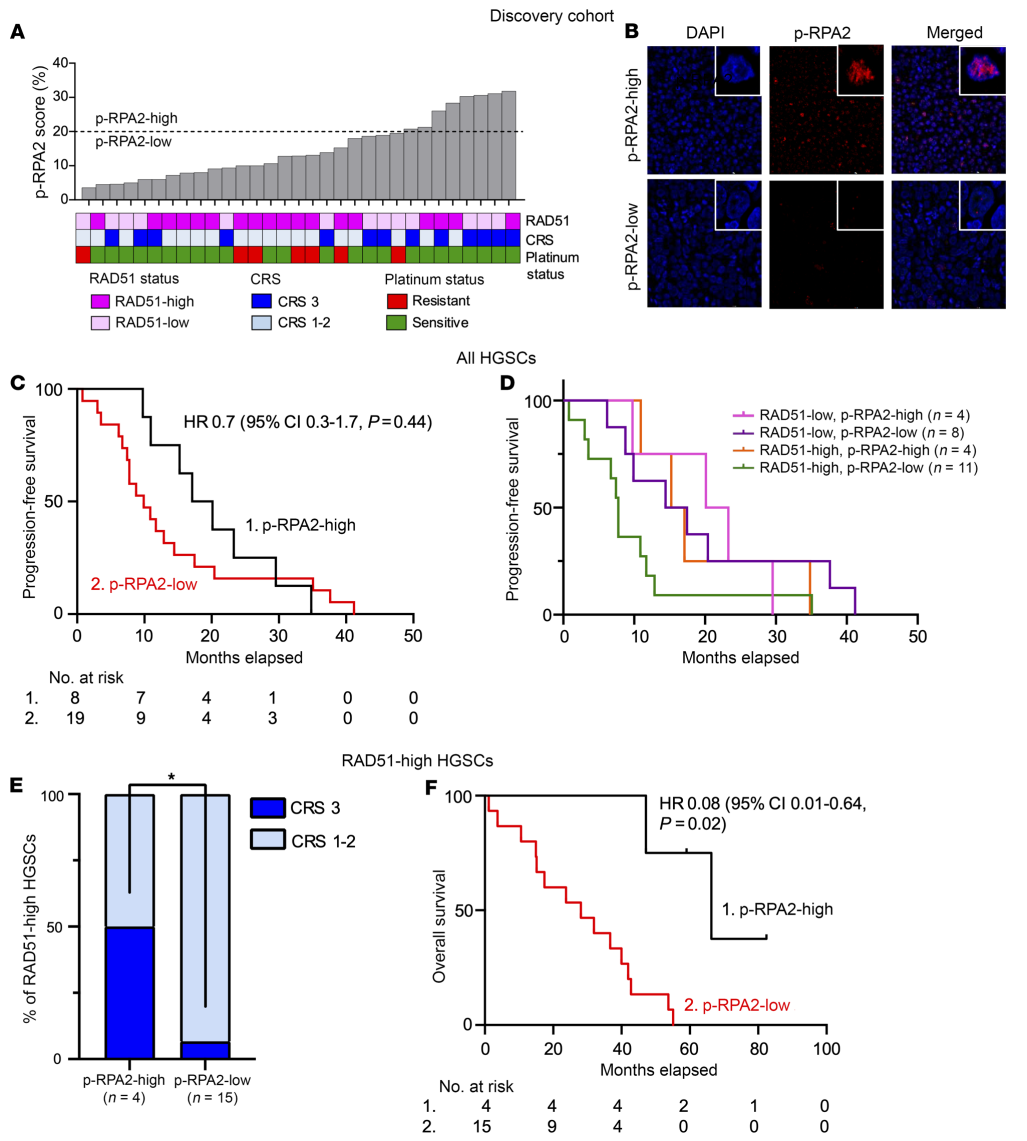
p-RPA2 score predicts therapy response and survival in patients with RAD51-high HGSCs treated with platinum chemotherapy in a discovery cohort. (**A**) RAD51 score, p-RPA2 score, chemotherapy response score (CRS), and platinum sensitivity in patients with HGSCs before neoadjuvant chemotherapy. p-RPA2 score is defined as the percentage of cells having ≥2 p-RPA2 foci. Dashed black line indicates manual quantification 20% cutoff, which delineates p-RPA2-high and p-RPA2-low HGSCs. All foci were counted in *n* > 100 cells per experiment. Technical replicates were performed for 30% of samples. (**B**) Representative images of DAPI and p-RPA2 and overlay of DAPI/p-RPA2 in patient-derived FFPE HGSC samples; images taken at ×63 original magnification. (**C**) Kaplan-Meier curves evaluating progression-free survival in patients with HGSCs stratified by p-RPA2 score (*n* = 27, hazard ratio 0.7, 95% CI 0.3–1.7, *P* = 0.4). (**D**) Kaplan-Meier curves evaluating progression-free survival in patients with HGSCs stratified by RAD51 and p-RPA2 scores. (**E**) Proportion of RAD51-high HGSCs that had a CRS of 3 stratified by p-RPA2 score (*P* = 0.03). (**F**) Kaplan-Meier curves evaluating overall survival in patients with RAD51-high HGSCs stratified by p-RPA2 score (*n* = 19, hazard ratio 0.08, 95% CI 0.01–0.64, *P* = 0.02). *P < 0.05, by Student’s 2-tailed *t* test.

All HGSCs had contributive results. p-RPA2 foci were manually counted in all HGSCs, and all samples had valid, quantifiable p-RPA2 foci (0–31 per cell). p-RPA2 foci were quantified by scoring of the percentage of cells with 2 or more p-RPA2 foci. Dichotomization cutoffs for the percentage of positive cells were defined by optimization of the overall survival (OS) and progression-free survival (PFS) difference in HR-proficient HGSCs (34) (Supplemental Figure 3). HGSCs were defined as p-RPA2-high if more than 20% of cells had at least 2 p-RPA2 foci and p-RPA2-low if ≤20% of cells had at least 2 p-RPA2 foci. Eight (25.8%) HGSCs were p-RPA2-high, and 23 (74.2%) were p-RPA2-low (Figure 1, A and B). Patients with p-RPA2-high HGSCs showed a trend toward increased PFS (17.1 vs. 9.9 months, hazard ratio 0.7, 95% CI 0.3–1.7, *P* = 0.4) and OS (47.1 vs. 32.7 months, hazard ratio 0.60, 95% CI 0.24–1.51, *P* = 0.3) compared with those with p-RPA2-low HGSCs (Figure 1C and Supplemental Figure 4). When p-RPA2 foci were evaluated in the context of HR status, the most profound survival differences were in patients with RAD51-high HGSCs. Patients with RAD51-high, p-RPA2-high HGSCs had longer PFS than patients with RAD51-high, p-RPA2-low HGSCs (15.2 vs. 7.8 months, hazard ratio 0.44, 95% CI 0.13–1.47, *P* = 0.18) (Figure 1D). Specifically, patients with RAD51-high, p-RPA2-high HGSCs had similar PFS to patients with RAD51-low HGSCs (15.2 vs. 17.5 months, hazard ratio 1.1, 95% CI 0.72–1.59, *P* = 0.73), suggesting that the level of replication stress may help to stratify survival in functionally HR-proficient HGSCs. Notably, this statistical analysis was limited by the cohort’s small sample size.

To determine the ability of p-RPA2 foci to directly predict platinum chemotherapy sensitivity in HR-proficient HGSCs, we assessed the association between p-RPA2 foci and chemotherapy response score (CRS) in RAD51-high HGSCs. A CRS is assigned to each HGSC at the time of interval cytoreductive surgery according to a validated histopathologic system and is a direct readout of platinum chemotherapy sensitivity (35). A CRS of 3 was consistent with a near pathologic complete response, and a score of 1–2 was considered a poor response to platinum chemotherapy. Thirteen (41.9%) HGSCs had a CRS of 3: 10 (83.3%) RAD51-low and 3 (15.8%) RAD51-high. Among the RAD51-low HGSCs, there were no differences in a CRS of 3 between p-RPA2-high and p-RPA2-low HGSCs (100% vs. 75%, *P* = 0.27) (Supplemental Figure 5A). Conversely, among RAD51-high HGSCs, p-RPA2-high HGSCs had significantly higher rates of a CRS of 3 than p-RPA2-low HGSCs (50% vs. 6%, *P* = 0.03) (Figure 1E). In this group, p-RPA2-high classification predicted a CRS of 3 with a specificity of 87.5% and a negative predictive value of 93.3%. Further, patients with RAD51-high, p-RPA2-high HGSCs had longer OS than those with p-RPA2-low HGSCs (66.3 vs. 28.1 months, hazard ratio 0.08, 95% CI 0.01–0.64, *P* = 0.02) (Figure 1F).

Because RAD51-high, p-RPA2-high HGSCs exhibited platinum sensitivity similar to that shown by RAD51-low HGSCs, we grouped RAD51-low and RAD51-high, p-RPA2-high HGSCs for further analysis. Patients with RAD51-low HGSCs and those with RAD51-high, p-RPA2-high HGSCs (*n* = 16) were significantly more likely to have a CRS of 3 than those with RAD51-high, p-RPA2-low HGSCs (*n* = 15) (74% vs. 6.7%, relative risk 11.3, 95% CI 1.7–76.3, *P* < 0.001) (Supplemental Figure 5B). The combined assay predicted a CRS of 3 with 92.3% sensitivity, 77.8% specificity, 93.3% negative predictive value, and 75.0% positive predictive value. When stage and *BRCA1/2* mutation status were controlled for, patients with RAD51-low or RAD51-high, p-RPA2-high HGSCs had significantly longer PFS (17.1 vs. 7.7 months, adjusted hazard ratio [aHR] 0.33, 95% CI 0.13–0.84, *P* = 0.02) (Supplemental Figure 5C) and OS (59.8 vs. 28.1 months, aHR 0.13, 95% CI 0.04–0.41, *P* < 0.001; Supplemental Figure 5D) than patients with RAD51-high, p-RPA2-low HGSCs.

**Figure 2:**
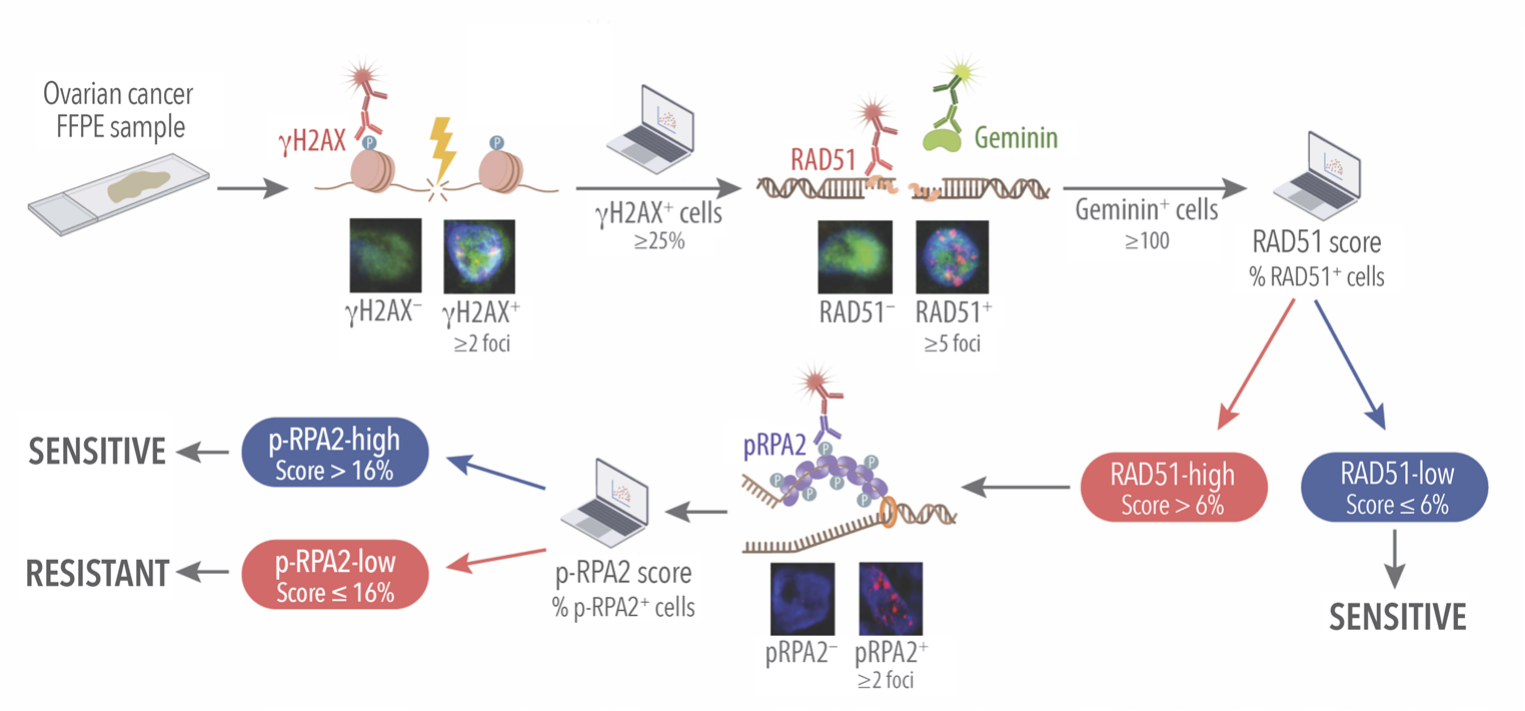
A functional replication stress assay for predicting response to DNA-damaging therapy in HR-proficient HGSCs. Schematic of combined RAD51 and p-RPA2 immunofluorescence assay in HGSC FFPE samples including automated quantification. After confirmation of cellularity in FFPE HGSC samples, they were screened for γH2AX. Only samples with 25% or more cells displaying 2 or more γH2AX foci were included in the analysis, ensuring sufficient DNA damage to elicit a DNA damage response. RAD51 foci were then assessed in geminin-positive cells, with cells containing 5 or more RAD51 foci classified as positive. Samples were considered RAD51-high or HR-proficient if more than 6% of the geminin-positive cells were positive for RAD51 on automated analysis. A minimum of 100 cells were assessed per sample. Subsequently, samples were evaluated for p-RPA2 foci. Cells with 2 or more p-RPA2 foci were considered positive, and samples with more than 16% of positive cells on automated analysis were considered p-RPA2-high or replication stress–high.

To facilitate translation of the RAD51, p-RPA2 assay into clinical care, we automated analysis of p-RPA2 foci (automated analysis of RAD51 assay was described in ref. 9). To do so, we used linear regression modeling to establish an automated p-RPA2 score cutoff that corresponded to the manual cutoff of 20%. We found strong correlation between a manual cutoff of 20% and an automated p-RPA2 cutoff of 16% (*r* = 0.7, *P* < 0.001; Cohen’s κ coefficient 0.72, *P* < 0.001). Using this cutoff, automated quantification had accuracy of predicting manual quantification of 96.6%, sensitivity of 100%, and specificity of 89.3%. Supplemental Figure 6 and Figure 2 illustrate the automated workflow and scoring criteria for our proposed combined HR and replication stress assay.

**Figure 3:**
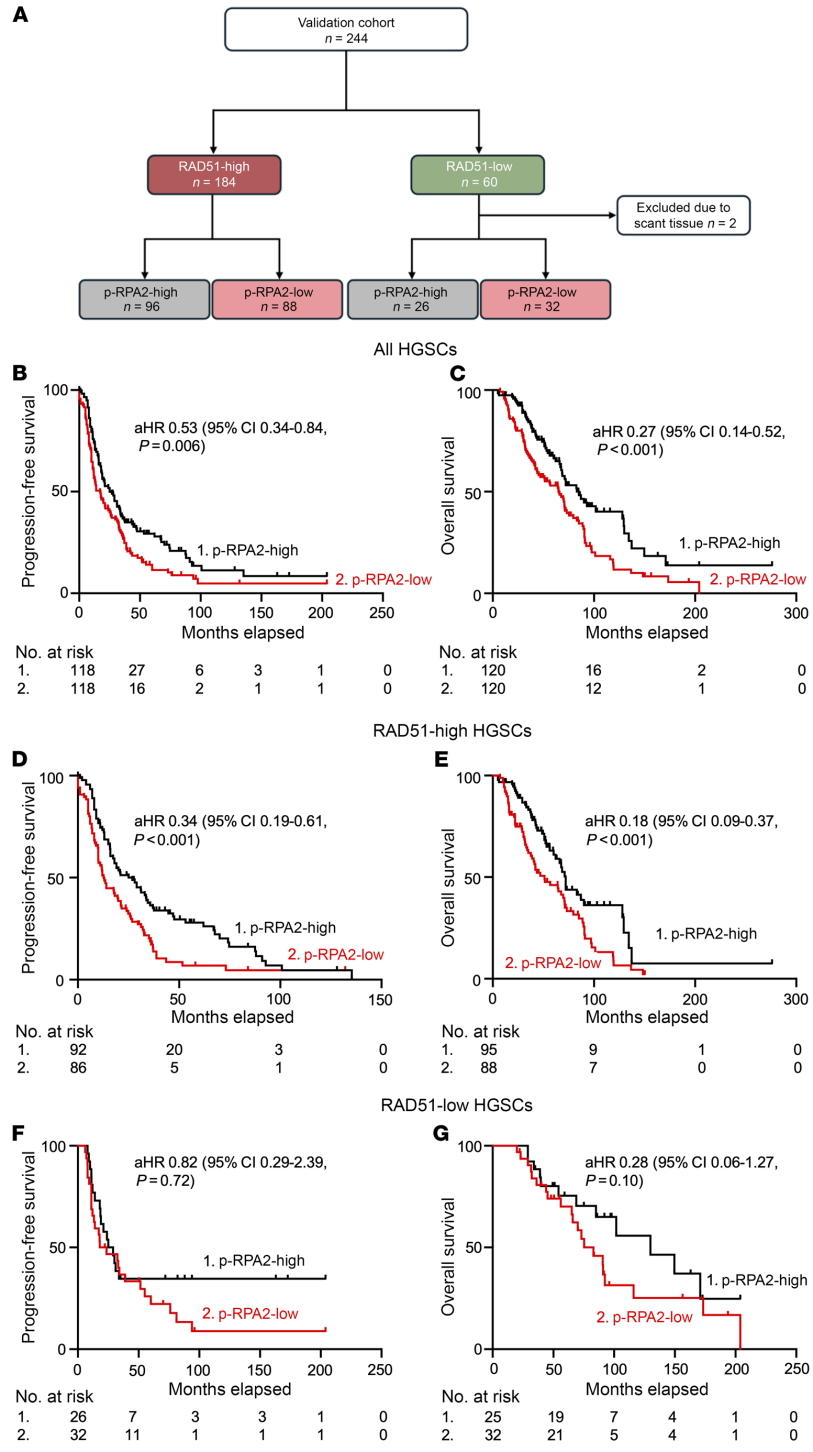
Automated p-RPA2 score predicts survival in patients with RAD51-high HGSCs treated with platinum chemotherapy in a validation cohort. (**A**) RAD51 and p-RPA2 scores in the validation cohort. (**B** and **C**) Kaplan-Meier curves evaluating progression-free survival (*n* = 236, aHR 0.53, 95% CI 0.34–0.84, *P* = 0.006) (**B**) and overall survival (*n* = 240, aHR 0.27, 95% CI 0.14–0.52, *P* < 0.001) (**C**) in patients with HGSCs stratified by p-RPA2 score. (**D** and **E**) Kaplan-Meier curves evaluating progression-free survival (*n* = 178, aHR 0.34, 95% CI 0.19–0.61, *P* < 0.001) (**D**) and overall survival (*n* = 183, aHR 0.18, 95% CI 0.09–0.37, *P* < 0.001) (**E**) in patients with RAD51-high HGSCs stratified by p-RPA2 score. (**F** and **G**) Kaplan-Meier curves evaluating progression-free survival (*n* = 58, aHR 0.82, 95% CI 0.29–2.39 *P* = 0.72) (**F**) and overall survival (*n* = 57, aHR 0.28, 95% CI 0.06–1.27, *P* = 0.10) (**G**) in patients with RAD51-low HGSCs stratified by p-RPA2 score. aHR, adjusted hazard ratio for age, stage, residual disease, and *BRCA* mutation status.

To validate the automated p-RPA2 foci assay to predict platinum responses in HR-proficient HGSCs, we screened an additional 246 high-grade serous ovarian, fallopian tube, or primary peritoneal cancers from patients receiving front-line platinum chemotherapy (Supplemental Figure 7). Two hundred thirty-eight (97.5%) samples evaluated were from primary and 8 (3.3%) were from metastatic HGSC. Two (0.8%) HGSCs were excluded because γH2AX scores were less than 25%, so 244 HGSCs were included in the final analysis (Table 1). All of these were HGSC, and the majority were advanced-stage disease (94.6%) and platinum-sensitive (86.9%). One hundred twenty-two (50.5%) were p-RPA2-high, and 120 (49.5%) were p-RPA2-low (Figure 3A). Of the 184 (75.4%) RAD51-high HGSCs, 96 (52.2%) were p-RPA2-high, and 88 (47.8%) were p-RPA2-low. On multivariate analysis controlling for stage, age, residual disease at the time of cytoreductive surgery, and *BRCA1/2* mutation status, patients with p-RPA2-high HGSCs had longer PFS (aHR 0.53, 95% CI 0.34–0.84, *P* = 0.006) (Figure 3B) and OS (aHR 0.27, 95% CI 0.14–0.52, *P* < 0.001) (Figure 3C) than patients with p-RPA2-low HGSCs. Upon further analysis, this effect seemed to be driven by the RAD51-high cases. Within the RAD51-high cases, patients with p-RPA2-high HGSCs had significantly longer PFS (26.9 vs. 12.7 months, aHR 0.34, 95% CI 0.19–0.61, *P* < 0.001) (Figure 3D) and OS (72.0 vs. 51.0 months, aHR 0.18, 95% CI 0.09–0.37, *P* < 0.001) (Figure 3E) than patients with p-RPA2-low HGSCs. Conversely, consistent with our findings in the discovery cohort, in RAD51-low HGSCs, p-RPA2 foci were not associated with PFS (aHR 0.82, 95% CI 0.29–2.39, *P* = 0.72) (Figure 3F) or OS (aHR 0.28, 95% CI 0.06–1.27, *P* = 0.10) (Figure 3G). These data suggest that replication stress is a major determinant of therapy sensitivity in HR-proficient HGSCs and can be implemented as a biomarker predictive of platinum chemotherapy response.

Upon image acquisition and data analysis, we noted heterogeneity in RAD51 and p-RPA2 staining. Therefore, to formally evaluate intratumoral heterogeneity within one site of disease, we compared RAD51 and p-RPA2 scores across at least 5 sites within each biopsy, analyzing a minimum of 100 cells per site and averaging the results for final classification. At diagnosis, RAD51 score exhibited a κ coefficient of 0.30 (95% CI 0.21–0.40, *P* < 0.001), indicating fair agreement across sampled regions within a biopsy. p-RPA2 score showed similar variability, with a κ coefficient of 0.26 (95% CI 0.16–0.35, *P* < 0.001), suggesting variability in regional expression.

We next assessed intratumoral heterogeneity between sites of disease. To do this we compared RAD51 and p-RPA2 scores between primary and metastatic sites (*n* = 112). RAD51 score demonstrated moderate concordance between primary and metastatic sites, with a κ coefficient of 0.50 (95% CI 0.31–0.68, *P* < 0.001). Among these, 16 (14.3%) were classified as RAD51-low, 77 (68.8%) as RAD51-high, and 19 (17%) exhibited heterogeneous staining. Similarly, p-RPA2 score exhibited moderate agreement between primary and metastatic HGSCs, with a κ coefficient of 0.42 (95% CI 0.11–0.74, *P* = 0.008). These findings indicate that despite variability in regional expression, RAD51 and p-RPA2 scores maintain moderate reproducibility between primary and metastatic sites, emphasizing the importance of analyzing multiple images per biopsy to ensure the sample is correctly classified.

To further explore the clinical implications of intratumoral heterogeneity, we evaluated PFS in patients with divergent RAD51 classification (RAD51-high or RAD51-low) between primary and metastatic samples, which we define as a divergent sample. We first compared PFS between patients with concordant RAD51-low, concordant RAD51-high, and divergent samples. While there was a trend toward decreased survival in patients with divergent HGSCs, the difference was not statistically significant, likely because of the small sample size. Therefore, we compared divergent samples with RAD51-high or RAD51-low samples within the entire cohort. Patients with divergent samples (*n* = 19) had significantly shorter PFS than those with RAD51-high (*n* = 163) or RAD51-low (*n* = 54) HGSCs (12.0 vs. 19.9 and 33.2 months, respectively, hazard ratio 1.56, 95% CI 0.19–2.04, *P* = 0.001) (Supplemental Figure 8A). Similarly, patients with divergent samples also had significantly shorter OS than those with RAD51-high or RAD51-low HGSCs (45.2 vs. 68.6 and 90.9 months, respectively, hazard ratio 1.64, 95% CI 1.3–2.30, *P* < 0.001) (Supplemental Figure 8B). This highlights the need for further investigation into intratumoral heterogeneity and its potential clinical implications.

To confirm the clinical applicability of the combined biomarker, we evaluated survival after grouping RAD51-low and RAD51-high, p-RPA2-high HGSCs. One hundred fifty-five (63.5%) were RAD51-low or RAD51-high, p-RPA2-high, and 89 (36.5%) were RAD51-high, p-RPA2-low. RAD51-low and RAD51-high, p-RPA2-high HGSCs were more likely to have sustained responses to platinum chemotherapy (PFS ≥ 12 months) than RAD51-high, p-RPA2-low HGSCs (71.1% vs. 44.8%, relative risk 1.59, 95% CI 1.23–2.04, *P* < 0.001). On multivariate analysis controlling for stage, age, residual disease at the time of cytoreductive surgery, and *BRCA1/2* mutation status, patients with RAD51-low or RAD51-high, p-RPA2-high HGSCs had significantly longer PFS (29 vs. 12 months, aHR 0.35, 95% CI 0.21–0.59, *P* < 0.001) (Supplemental Figure 9A) and OS (83 vs. 47 months, aHR 0.18, 95% CI 0.09–0.35, *P* < 0.001) (Supplemental Figure 9B) than patients with RAD51-high, p-RPA2-low HGSCs.

### RAD51 and p-RPA2 foci predict survival outcomes in patients with RAD51-high HGSCs treated with PARP inhibitors.

**Figure 4:**
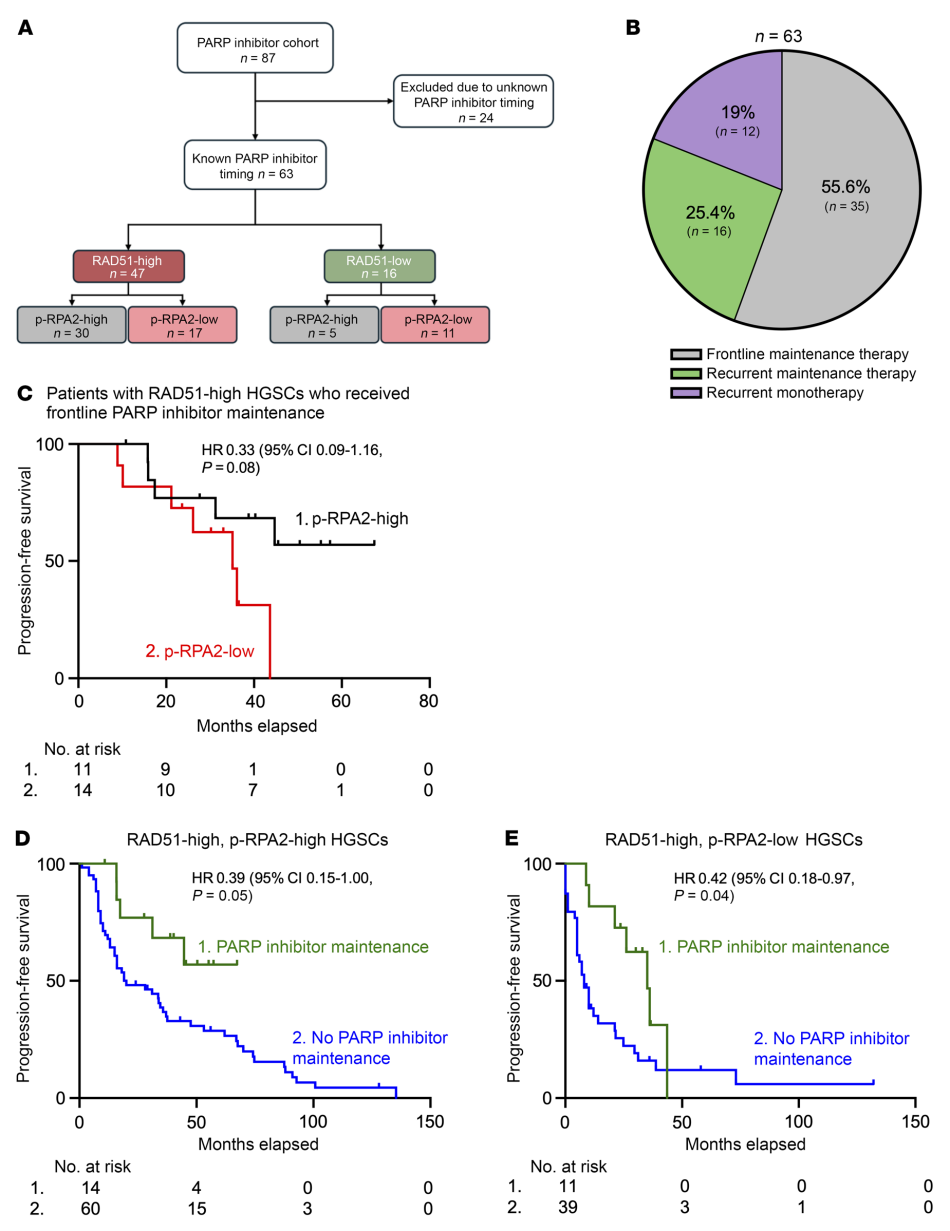
Automated p-RPA2 score predicts survival in patients with RAD51-high HGSCs treated with PARP inhibitors. (**A**) RAD51 and p-RPA2 scores of patients treated with a PARP inhibitor. (**B**) Pie chart illustrating the timing of PARP inhibitor therapy. (**C**) Kaplan-Meier curves evaluating progression-free survival in patients with RAD51-high HGSCs who received frontline PARP inhibitor therapy stratified by p-RPA2 score (*n* = 25, hazard ratio 0.33, 95% CI 0.09–1.16, *P* = 0.08). (**D** and **E**) Kaplan-Meier curves evaluating progression-free survival in patients with RAD51-high, p-RPA2-high HGSCs (*n* = 74, hazard ratio 0.39, 95% CI 0.15–1.00, *P* = 0.05) (**D**) or RAD51-high, p-RPA2-low HGSCs (*n* = 50, hazard ratio 0.42, 95% CI 0.18–0.97, *P* = 0.04) (**E**) treated with or without frontline PARP inhibitor maintenance therapy.

We next wanted to determine the utility of p-RPA2 foci for predicting survival after PARP inhibitor treatment in RAD51-high HGSCs. We analyzed samples from a cohort of 87 ovarian cancer patients who received PARP inhibitors at any point during their treatment course (Table 1 and Figure 4A). Twenty-four (27.6%) samples were excluded because the timing of PARP inhibitor treatment was unknown. Thirty-five (55.6%) patients received PARP inhibitors as frontline maintenance therapy, 16 (25.4%) as recurrent maintenance therapy, and 12 (19.0%) as recurrent monotherapy (Figure 4B). Forty-seven (74.6%) HGSCs were RAD51-high. Of these, 30 (63.8%) were p-RPA2-high and 17 (36.2%) were p-RPA2-low. In the initial PFS analysis, we only included the 25 patients with RAD51-high HGSCs who received PARP inhibitors as frontline maintenance therapy. In these 25 patients, those with p-RPA2-low HGSCs had a median PFS of 35.1 months, whereas those with p-RPA2-high HGSCs did not meet a median survival (64.3% of cases were censored because they had not yet recurred) (hazard ratio 0.33, 95% CI 0.09–1.16, *P* = 0.08) (Figure 4C). This difference was large, but not statistically significant, as the data are not yet mature.

We next examined PFS in the 14 patients with RAD51-high HGSCs who received PARP inhibitor maintenance therapy in the recurrent setting, recognizing the limitations of this analysis due to the small sample size and inability to control for confounding variables. Patients with p-RPA2-high HGSCs at diagnosis had significantly longer PFS after PARP inhibitor therapy than those with p-RPA2-low HGSCs (14.5 vs. 2.4 months, hazard ratio 0.04, 95% CI 0.01–0.42, *P* = 0.006) (Supplemental Figure 10A). Lastly, we examined PFS after PARP inhibitor therapy in the 8 patients with RAD51-high HGSCs who received PARP inhibitor monotherapy in the recurrent setting. Similarly, patients with p-RPA2-high HGSCs had longer PFS than those with p-RPA2-low HGSCs (15.3 vs. 3.0 months, hazard ratio 0.36, 95% CI 0.05–2.59, *P* = 0.3) (Supplemental Figure 10B). Notably, these biopsies are archival, and immediate pretreatment biopsies would be more ideal for a more direct evaluation of the relationship between p-RPA2 status and PARP inhibitor response. To further understand the predictive potential of this biomarker, we analyzed PFS in patients with RAD51-high HGSCs who did and did not receive frontline PARP inhibitor maintenance therapy stratified by p-RPA2 status. First, we evaluated patients with RAD51-high, p-RPA2-high HGSCs. At 36 months follow-up, 42.8% (6/14) of patients who received PARP inhibitors experienced a recurrence. In contrast, 68.3% (41/60) of patients who did not receive PARP inhibitor maintenance recurred at the same time point. PARP inhibitor maintenance in these patients was associated with longer PFS (undefined vs. 20.0 months, hazard ratio 0.39, 95% CI 0.15–1.00, *P* = 0.05) (Figure 4D) and a trend toward OS (82.5 vs. 67.6 months, hazard ratio 0.28, 95% CI 0.03–1.35, *P* = 0.09) (Supplemental Figure 10C). Notably, in the OS analysis, 92.9% of patients who received PARP inhibitor maintenance were censored. Next, we analyzed RAD51-high, p-RPA2-low HGSCs. At 36 months follow-up, 72.7% (8/11) of patients with RAD51-high, p-RPA2-low HGSCs who received PARP inhibitors experienced a recurrence. Conversely, 87.1% (34/39) of patients who did not receive PARP inhibitor maintenance had recurred at the same time point. PARP inhibitor maintenance in these patients was associated with increased PFS (35.1 vs. 8.0 months, hazard ratio 0.42, 95% CI 0.18–0.97, *P* = 0.04) (Figure 4E), but not with OS (37.6 vs. 33.0 months, hazard ratio 0.26, 95% CI 0.19–1.57, *P* = 0.26) (Supplemental Figure 10D). Markedly, the median PFS was significantly longer in patients with RAD51-high, p-RPA2-high HGSCs treated with PARP inhibitors than in patients with p-RPA2-low HGSCs. Together, these data suggest that PARP inhibitor maintenance may benefit patients with RAD51-high, p-RPA2-high HGSCs.

### RAD51 and p-RPA2 foci predict patient survival in recurrent HGSCs.

**Figure 5:**
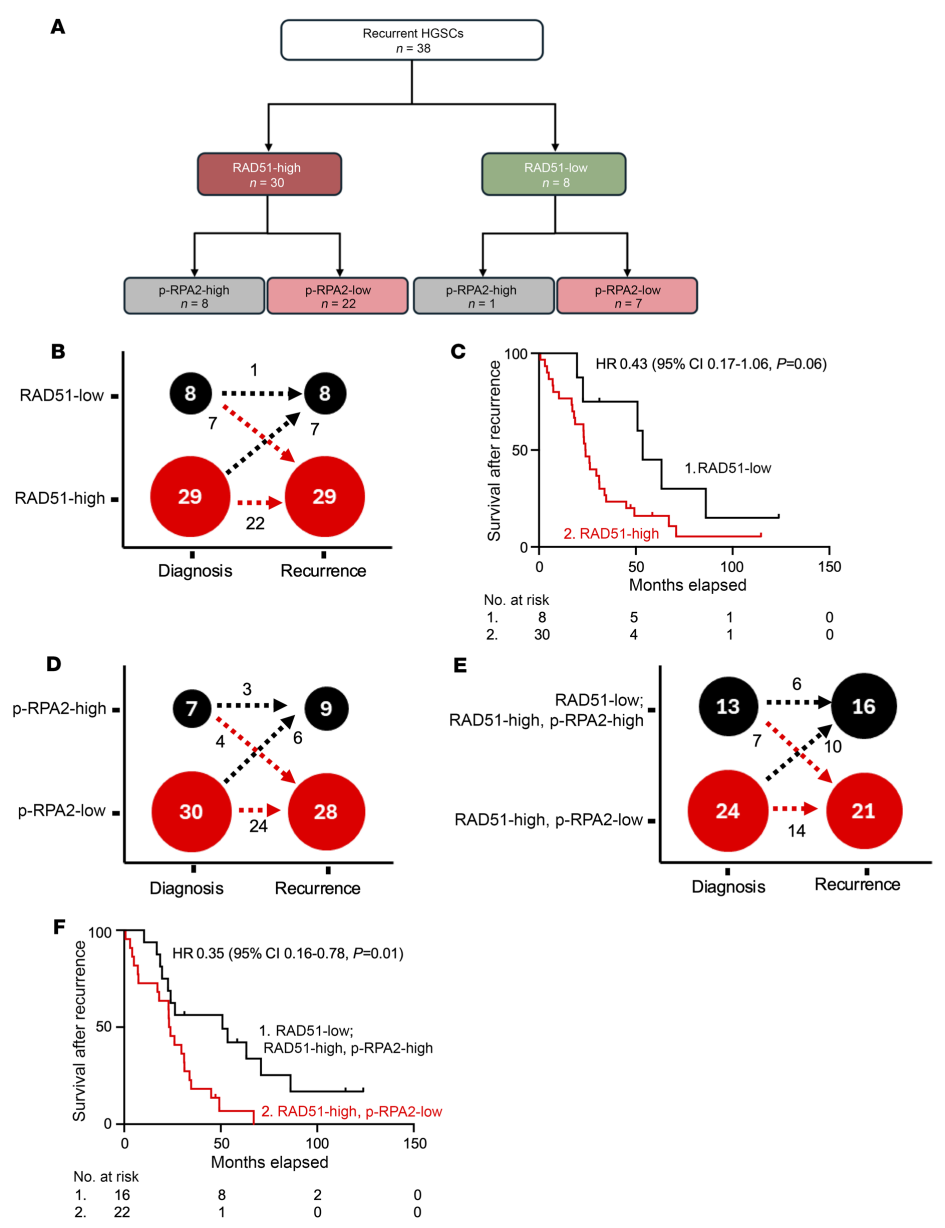
Automated p-RPA2 and RAD51 scores at the time of recurrence predict survival. (**A**) RAD51 and p-RPA2 scores in recurrent HGSCs. (**B**) HGSCs stratified by RAD51 score at time of diagnosis and recurrence (*n* = 37). (**C**) Kaplan-Meier curves evaluating overall survival after recurrence in patients with HGSCs stratified by RAD51 score at time of recurrence (*n* = 38, hazard ratio 0.43, 95% CI 0.17–1.06, *P* = 0.06). (**D**) HGSCs stratified by p-RPA2 score at time of diagnosis and recurrence (*n* = 37). (**E**) HGSCs stratified by RAD51 and p-RPA2 scores at time of diagnosis and recurrence (*n* = 37). (**F**) Kaplan-Meier curves evaluating overall survival after recurrence in patients with HGSCs stratified by RAD51 and p-RPA2 scores at time of recurrence (*n* = 38, hazard ratio 0.35, 95% CI 0.16–0.78, *P* = 0.01).

A major limitation of currently available genomic HR assays is their static nature. The genomic instability that results from HR-deficient DNA repair mechanisms is not reversible, so a cancer will be defined by genomic scar assays as HR-deficient even if it evolves to be HR-proficient (36–38). In our assay, RAD51 and p-RPA2 staining only produces foci if the cancer cells recently experienced HR and replication stress, thereby providing a dynamic reading of both processes. To test this idea, we evaluated HGSC RAD51 and p-RPA2 scores in 42 patients from whom we had recurrent HGSC biopsies. Of these, HGSCs from 4 (9.5%) patients had γH2AX scores less than 25% and were excluded from the analysis. Of the remaining 38 patients, 37 had matched HGSC samples from diagnosis and recurrence (Figure 5A and Supplemental Figure 7). At the time of diagnosis, 29 (76.3%) HGSCs were RAD51-high and 8 were RAD51-low (21.1%) (Figure 5B). At the time of recurrence, 14 (37.8%) HGSCs changed RAD51 classification. Patients with RAD51-low HGSCs at recurrence had longer survival after recurrence than those with RAD51-high HGSCs (53.5 vs. 23.9 months, hazard ratio 0.43, 95% CI 0.17–1.06, *P* = 0.06) (Figure 5C). Next, we considered p-RPA2 score. Ten (27.0%) patients had a change in their p-RPA2 classification between diagnosis and recurrence (Figure 5D). At the time of recurrence, 9 (24.3%) were p-RPA2-high and 28 (75.7%) were p-RPA2-low (Figure 5D). To improve the power of this analysis, we next considered RAD51-low and RAD51-high, p-RPA2-high HGSCs together. Twenty (54.0%) HGSCs had the same classification at diagnosis and recurrence, and 17 (45.9%) were reclassified (Figure 5E). Patients with RAD51-low or RAD51-high, p-RPA2-high HGSCs at recurrence had longer OS (50.9 vs. 23.2 months, hazard ratio 0.35, 95% CI 0.16–0.78, *P* = 0.01) than those with RAD51-high, p-RPA2-low HGSCs (Figure 5F). We conclude that the RAD51, p-RPA2 assay is dynamic and useful at time of recurrence.

## Discussion

While nearly all HR-deficient HGSCs are sensitive to therapy, a subset of HR-proficient HGSCs also exhibit therapeutic sensitivity (9). Here, we demonstrate that a key determinant of response in HR-proficient HGSCs is replication stress. To date, no functional biomarkers of replication stress have been developed for use on clinically available specimens. Our work describes the development and validation of a functional assay to measure replication stress in FFPE HGSCs. We first demonstrate that we can reliably evaluate replication stress in FFPE cancer cells with an antibody targeting Thr21-phosphorylated RPA2 (p-RPA2). Next, in a discovery cohort, we show that RAD51-high (HR-proficient) HGSCs with high p-RPA2 respond to platinum chemotherapy similarly to RAD51-low (HR-deficient) HGSCs. We then used a validation cohort to demonstrate that patients with RAD51-high, p-RPA2-high HGSCs had longer PFS and OS after platinum chemotherapy than patients with RAD51-high, p-RPA2-low HGSCs. Additionally, our data suggest that p-RPA2 foci may accurately identify which RAD51-high HGSCs will and will not respond to PARP inhibitors. Finally, we showed that the assay is dynamic and predictive in recurrent HGSCs.

Several lines of published and preliminary evidence indicate that increasing replication stress can sensitize HR-proficient HGSCs to DNA-damaging therapy. First, replication stress–inducing agents, such as inhibitors of ATR, WEE1, and CHK1/2, are being explored in clinical trials with promising early results specifically in HR-proficient cancers (39–42). Second, in a phase II clinical trial in platinum-resistant ovarian cancer evaluating the combination of the replication stress–inducing agents gemcitabine and an ATR inhibitor, ovarian carcinomas with high replication stress defined by genomic measures responded better to combination therapy independent of HR status (39). Third, multiple studies have evaluated the combination of gemcitabine and carboplatin in platinum-sensitive and platinum-resistant ovarian cancer. They have demonstrated impressive response rates of over 75% in platinum-sensitive cancer and 65% in platinum-resistant cancer, with survival greater than 25 months in select platinum-resistant patients (43–48). This is remarkable for a cohort of patients who typically have response rates to subsequent chemotherapy of 15% and survival of 12 months. Although these studies have proposed targeting replication stress, a standardized method for measuring it is currently lacking. Our assay offers a validated workflow for measuring replication stress.

Although our data illustrate a clear association between therapy response and p-RPA2 foci, the mechanisms driving high p-RPA2 in some cancers are not fully elucidated. High numbers of p-RPA2 foci could reflect increased replication fork stalling, fork degradation, or ssDNA gaps. Fork stalling can result from DNA damage or replication obstacles. Upon encountering a DNA lesion, the replication polymerase decouples from the helicase, promoting DNA unwinding and accumulation of ssDNA between the polymerase and the helicase. This ssDNA is coated by RPA (25), which protects the ssDNA from nucleolytic degradation. Cells that can resolve this stalling and restart replication are often therapy resistant (49). Upon stalling, the replication fork can reverse. In this configuration, the complementary leading and lagging nascent strands of DNA anneal, creating a “chicken foot” structure (27). This mechanism relies on the ability of cells to protect the newly synthesized DNA by loading RAD51. If the cell cannot protect the DNA, nucleases degrade the DNA, leaving behind ssDNA that is coated with RPA. Replication fork degradation is associated with sensitivity to chemotherapy in established ovarian cancer cell lines (28). Alternatively, to overcome fork stalling, the replisome can skip the obstructing lesion, reprime the DNA with PRIMPOL, and continue replication. This results in an ssDNA gap, which is coated with RPA. Increased gaps can also be a result of defective Okazaki fragment processing or defective gap repair by translesion synthesis or template switching. Replication ssDNA gaps have been proposed to drive therapy sensitivity independent of HR status in cell lines (23, 24, 29). It is possible that increased p-RPA accumulation can be a cumulative event from varied mechanisms of replication fork remodeling. Further work is necessary to determine which events are responsible for therapy response in HR-proficient HGSCs.

An important finding of our study is that the RAD51, p-RPA2 assay might be useful for recurrent HGSC samples. HR status is currently assessed clinically by germline or somatic mutation testing or evaluation of genomic scars. However, these assays all provide a snapshot of past events and do not necessarily reflect the current HR capacity of the cancer. Clinical ability to predict whether cancer will respond to platinum at recurrence is also poor. For example, historical data suggest that cancers in up to 50% of patients in a “platinum-resistant” cohort respond to further platinum treatment (50). Conversely, cancers in only about 50%–60% of patients with disease-free intervals greater than 6 months — considered “platinum-sensitive” — respond to repeat platinum-based therapy (19). Therefore, over 50% of patients have misclassified platinum sensitivity and either are not offered the most effective drug for their disease or are given an ineffective, toxic therapy (50). With additional validation, the RAD51, p-RPA2 assay described here may allow clinicians to expand chemotherapy to patients with recurrent disease and improve their long-term outcomes.

Another important finding of our study is the intratumoral heterogeneity observed in RAD51 and p-RPA2 foci staining both within one biopsy and between different sites of disease. This is likely driven by increased clonal diversity, resulting in variable DNA repair proficiency across different subclones. Consistent with previous findings that greater clonal diversity has been associated with a higher likelihood of therapy-resistant and metastatic subpopulations (51, 52), we found that patients with heterogeneous RAD51 and p-RPA2 staining in their cancers exhibited the worst survival outcomes. It is also possible that heterogeneity was exaggerated as a result of the inclusion of stromal or immune cells, as we did not use an epithelial marker to specifically identify tumor cells in the analysis. Notably, a subset of RAD51-high HGSCs at diagnosis retained persistent RAD51-low subclones at recurrence. All of these samples were obtained at first recurrence, and all HGSCs were platinum sensitive. While platinum chemotherapy is expected to eliminate HR-deficient subclones, these findings suggest the emergence of resistance mechanisms independent of HR. Potential survival pathways include reliance on alternative end-joining repair (e.g., non-homologous or microhomology-mediated end joining), increased replication fork stabilization, upregulation of drug efflux transporters, or tumor microenvironmental changes supporting residual cancer cell survival. These findings underscore the adaptive plasticity of HR-deficient HGSCs under selective therapeutic pressure and highlight the clinical significance of intratumoral heterogeneity in disease progression and treatment response.

Our work has several limitations. First, although automated, our assay still requires a trained technician to image the stained microscopy slides. Second, our assay uses deconvolution imaging, which might not be readily available. Third, we did not have homologous recombination deficiency (HRD) status from commercial assays based on allelic imbalance assays to compare with our RAD51 status. Thus, we were unable to directly compare the performance of the RAD51, p-RPA2 assay with those of clinically used methods for assessing HRD status. This is a future aim of our work. Fourth, the survival data for patients who received PARP inhibitors were not mature, so the differences described are likely underestimated. Fifth, we observed intratumoral heterogeneity in RAD51 and p-RPA2 foci staining within HGSC samples, with the greatest variability observed within individual samples. This heterogeneity was reduced when the final classification of primary versus metastatic HGSCs was compared. This suggests that analyzing at least 5 representative images per sample can mitigate some of this variability. Given that most samples analyzed were from primary HGSCs, our findings support prioritizing primary samples for evaluation when feasible. Additionally, analyzing multiple distinct carcinoma sites (e.g., primary and metastatic samples) will likely further address this heterogeneity. Nonetheless, intratumoral heterogeneity likely affects the final classification of HGSCs and, based on our preliminary findings, may have substantial clinical implications. Further research is needed to understand how this heterogeneity should impact clinical decision-making. Sixth, samples were retrospectively collected from patients with varying treatment regimens across different institutions. These results should be validated in a controlled cohort, preferably HGSC samples from a clinical trial. And finally, while this assay predicts therapy resistance, it does not propose alternative treatment regimens. However, this work does establish a clear mechanism of sensitivity in HR-proficient HGSCs that can be targeted to overcome resistance. For example, perhaps patients with p-RPA2-low HGSCs should be treated with platinum chemotherapy in combination with a replication stress–inducing agent such as gemcitabine. On the other hand, those with p-RPA2-high HGSCs will respond well to standard-of-care platinum chemotherapy and paclitaxel. Future work will clarify this.

In conclusion, when combined with HR proficiency, our automated p-RPA2 assay can be a powerful clinical tool for predicting response to platinum chemotherapy and PARP inhibitors. Its dynamic nature allows for real-time cancer behavior evaluation, making it particularly valuable in managing recurrent disease. Further prospective studies are needed to validate these findings, compare the assay’s predictive performance with clinically available genomic measures, and facilitate its integration into routine clinical practice.

## Methods

### Sex as a biological variable.

Our study exclusively examined females because ovarian cancer is only relevant in females.

### Patient samples.

For the discovery cohort, tissue was collected prospectively from patients with stage III–IV high-grade serous ovarian cancer before neoadjuvant chemotherapy as part of a national clinical trial (IRB 201407156). All tissues were collected between December 2014 and December 2018. Chemotherapy response score was assigned at the time of interval cytoreductive surgery according to a validated system (53). A chemotherapy response score of 3 was consistent with a near pathologic complete response, and a score of 1–2 was considered a poor response to platinum chemotherapy. PFS and OS were calculated from the time of interval cytoreductive surgery.

For the validation cohort, 4 tissue microarrays of HGSC samples were used. The first consisted of deidentified samples from the University of Kansas. The second and third were built in Anatomic and Molecular Pathology Core Laboratories at Washington University (IRB 202301067). These both contained primary and/or metastatic HGSCs collected during primary or interval cytoreductive surgery after chemotherapy. The fourth, from Cedars-Sinai Medical Center, contained deidentified matched primary, metastatic, and recurrent HGSC samples (IRB Pro44852) (54).

### Development of primary ovarian cancer cells and cell culture.

Tissues were prospectively collected for our Gynecologic Oncology Biorepository (IRB 201105400 and 201706151) with written informed patient consent. To establish primary cell lines, ascites was collected from patients with advanced-stage serous ovarian cancer and transferred to culture flasks containing 1:1 (vol/vol) RPMI medium supplemented with 20% FBS and 1% penicillin and streptomycin. After 1–2 weeks, attached and proliferating cells were passaged and used for experiments. Cells were discarded after 1 to 2 passages.

### Immunofluorescence.

Primary ovarian cancer cells (40,000 cells per well) were plated in 8-well chamber slides. Cells were washed with cold PBS, fixed for 10 minutes with 2% paraformaldehyde, permeabilized for 20 minutes with 0.2% Triton X-100 in PBS, and washed again with PBS. Cells were blocked for 30 minutes in PBS, 0.5% BSA, 0.15% glycine, and 0.1% Triton X-100 and incubated overnight at 4°C with primary antibodies (Supplemental Table 1) in staining buffer. Cells were then stained with secondary antibodies (Supplemental Table 1), followed by DAPI (MilliporeSigma). Cells were imaged on a Leica TCS SPE inverted confocal microscope. Raw images were exported, and JCountPro was used to count the foci (9, 55, 56). At least 100 cells were analyzed for each treatment group in duplicate.

### Transfection.

TYKNU and OVCAR8 cells were transfected with RPA2 Silencer Select siRNA (Thermo Fisher Scientific) or non-targeting control siRNA (Thermo Fisher Scientific) using Lipofectamine RNAiMAX transfection reagent (Thermo Fisher Scientific) according to the manufacturer’s instructions. Two days after transfection, cells were treated with hydroxyurea (MilliporeSigma).

### FFPE cell blocks.

The cells were fixed in 4% paraformaldehyde for 1 hour. The cell pellet was embedded in 2% agarose and put into a cassette in 10% formalin for an additional 24 hours. The cell pellets were washed with deionized water and dehydrated in sequential concentrations of ethanol (30%, 50%, and 70%). The samples were embedded in paraffin wax, cut into 4-mm sections, and mounted onto slides for further analysis.

### Western blot analysis.

Cells transfected with siRPA2 or non-targeting siRNA (siControl) were lysed in 9 mol/L urea, 0.075 mol/L Tris, pH 7.6, and protein concentration was determined using the Bradford assay (Bio-Rad). Protein lysates (60–100 μg) were separated on SDS-PAGE, transferred to nitrocellulose, probed with primary antibody (Supplemental Table 1) at 4°C overnight in humid chambers, washed, and incubated with corresponding horseradish peroxidase–conjugated secondary antibodies (Jackson ImmunoResearch). Pierce ECL Western Blotting Substrate (Thermo Fisher Scientific) was used to detect signal, and chemiluminescence was measured on a ChemiDoc (Bio-Rad Laboratories).

### MTS cell viability assay.

Cells were seeded at 2,000 cells per well in a 96-well plate. After 24 hours, cells were treated with a range of carboplatin (Teva Pharmaceuticals). Viability was assessed 6 days after treatment using an MTS/PMS (Promega PR-G1112) solution and absorbance read at 490 nm on an Infinite M200 Pro plate reader (Tecan Inc.). IC_50_ values were calculated in GraphPad Prism (GraphPad Software Inc.).

### FFPE immunofluorescence.

Immunofluorescence on FFPE samples was completed as previously described (9). Briefly, hematoxylin and eosin–stained slides were examined by a board-certified pathologist to assess cellularity and diagnosis. Corresponding unstained slides (4-μm-thick sections) were deparaffinized, dehydrated, and stained with primary and secondary fluorescent antibodies (Supplemental Table 1). Samples were stored at –20°C. Imaging was performed on a Leica DMi8 microscope with Thunder Imaging computational clearing.

A dichotomization cutoff optimization method was used to determine the p-RPA2 cutoff (≥20% of cells with ≥2 foci). To ensure robustness, we applied a systematic statistical approach, evaluating multiple potential cutoffs and selecting the one that provided the most significant stratification of PFS and OS outcomes (Supplemental Figure 3, A and B).

### Automated foci analysis.

Stained microscopy images were imported into the R environment. After denoising, smoothing, and thresholding, a 2-dimensional convolution was applied to segment all the foci in the image. Then, the foci-positive cells were counted, and the ratio of foci-positive cells to all cells was calculated. With multiple images from each patient (*n* ≥ 5), a mean ratio was computed to estimate the γH2AX, RAD51, and p-RPA2 scores. For the automated cutoff value, we established a linear relationship between the manual p-RPA2 score and the automated p-RPA2 score using a linear regression model. The regression coefficient was then used to determine an automated p-RPA2 cutoff that corresponds to the manual 20% threshold.

### Sample size calculation.

The sample size necessary to detect a significant hazard ratio of PFS from the patients with RAD51-high, p-RPA2-high HGSCs compared with the patients with RAD51-high, p-RPA2-low HGSCs (57, 58) was calculated. Our discovery cohort showed a ratio of unexposed to exposed around 1, the baseline of probability of event around 90%. If the expected hazard ratio of RAD51-high, p-RPA2-high HGSCs was less than 0.44 based conservatively on our preliminary data, then 58 patients would provide the study with 80% power or 70 patients with 90% power, at a 2-sided significance level of 0.05. Therefore, the validation cohort of 244 patients was highly adequate to assess the predictive value of the RAD51, p-RPA2 assay.

### Generative AI program use.

We used ChatGPT (version 4o) between January 2025 and June 2025. for minor grammar edits during manuscript preparation.

### Statistics.

Traditional statistical analyses were performed in GraphPad Prism 9 and SPSS version 27 software (9). Baseline patient demographics were assessed with descriptive statistics, excluding any missing data from the analysis. Independent Student’s 2-tailed *t* tests and Mann-Whitney *U* tests were used to compare continuous variables. One-way ANOVA was applied where appropriate. For correlation analyses, the Spearman’s rank-order correlation coefficient was used owing to small size and potential non-normality of the data. Poisson regression was used to determine relative risks. PFS and OS were measured from date of surgery to date of evidence of disease recurrence, death, or last contact if no recurrence was observed. Patients without recurrence or still alive were censored at their last contact date. The Kaplan-Meier method estimated survival times, with comparisons made via the log-rank test. Cox proportional-hazards regression was done in univariate and multivariate formats as needed. For multivariate Cox proportional-hazards model analyses we controlled for stage, age, residual disease, and/or *BRCA1/2* mutation status based on their established clinical importance in ovarian cancer prognosis. These variables are well-recognized predictors of patient outcomes and are commonly included in similar analyses. We assessed proportional-hazards assumption using Schoenfeld residual tests (Supplemental Figure 11, A–D). Missing data were handled by SPSS through listwise deletion, and no imputation was performed. Censoring was handled using a standard approach, where patients were censored if they had not experienced the event (e.g., recurrence or death) by the end of the follow-up period. While we did not perform a formal comparison of censored versus event-experiencing patients, there is no indication that censored individuals systematically differed in key clinical characteristics. However, we acknowledge this as a potential limitation and recognize that unmeasured factors could influence censoring patterns. Intratumoral heterogeneity was assessed using Fleiss’ κ to evaluate agreement across multiple measurements within a single biopsy site, and Cohen’s κ was used to evaluate agreement between primary and metastatic sites.

Statistical significance was set at *P* less than 0.05, with 2-tailed 95% confidence intervals (CIs). Bonferroni’s correction was applied where appropriate to account for multiple comparisons and control type I error inflation.

### Study approval.

This research was approved for the use of human subjects by the Washington University in St. Louis, Missouri Institutional Review Board (IRB #201407156, #202301067) and the Cedars-Sinai Medical Center in Los Angeles, California Institutional Review Board (IRB Pro44852). All study procedures involving human specimens were conducted in accordance with the ethical standards of the respective IRBs.

### Data availability.

The data produced in this study can be made available upon request. Values for all data points in graphs are reported in the Supporting Data Values file.

## Author contributions

AS and AC conceptualized experiments, conducted experiments, acquired data, analyzed data, and wrote, edited, and reviewed the manuscript. RD, ML, KR, and JB acquired data, edited the manuscript, and provided critical feedback and edits on the manuscript. SH provided patient samples and edited the manuscript. CS acquired data and edited the manuscript. BS, LK, CM, ARH, PT, DM, and MP provided patient samples and critical feedback and edits on the manuscript. VS provided experimental design advice and provided critical feedback and edits on the manuscript. ISH evaluated patient samples and provided critical feedback and edits on the manuscript. AEW, BYK, SO, and KCF provided patient samples and provided critical feedback and edits on the manuscript. LS analyzed and confirmed pathology of samples and provided critical feedback and edits on the manuscript. PV conceptualized and designed experiments and provided critical feedback and edits on the manuscript. EL conceptualized experiments, conducted experiments, acquired data, analyzed data, and wrote and edited the manuscript. PZ wrote code for automatic quantification of foci, conducted statistical analyzes, and provided critical feedback and edits on the manuscript. DK provided patient samples, experimental design feedback, and critical feedback and edits on the manuscript. MMM provided funding and reagents, conceptualized experiments, conducted experiments, acquired data, analyzed data, and wrote and edited the manuscript.

## Supplementary Material

Supplemental data

ICMJE disclosure forms

Unedited blot and gel images

Supporting data values

## Figures and Tables

**Table 1:**
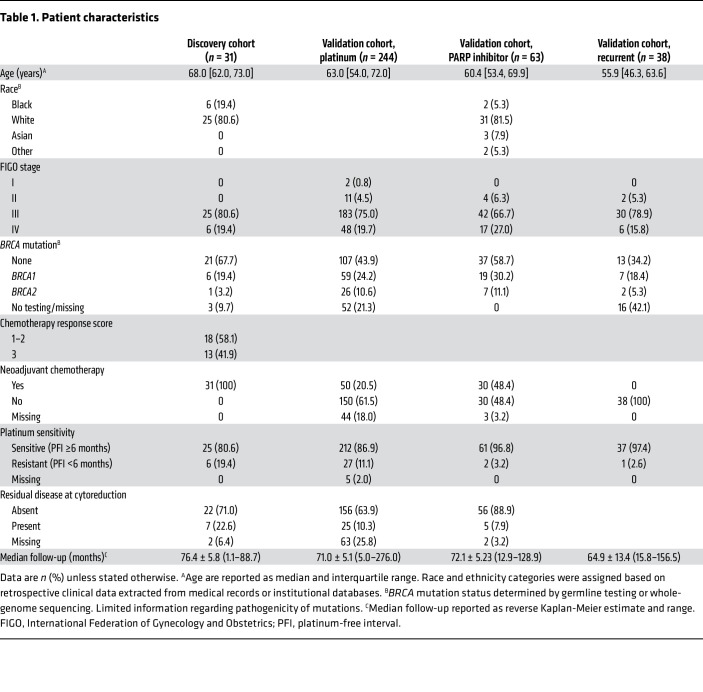
Patient characteristics

## Version 1 05/20/2025

In-Press Preview

## Version 2 07/01/2025

Electronic publication

## Version 3 07/03/2025

Corrected labels in the graphical abstract

## Version 4 07/14/2025

corrected graphical abstract title
