# Supplementary material for: Phospho-RPA2 predicts response to platinum and PARP inhibitors in homologous recombination–proficient ovarian cancer
Source: J Clin Invest. 2025 May 20;135(13):e189511. doi: 10.1172/JCI189511 (PMC12208538; doi:10.1172/JCI189511)
Supplement: Supplemental data [file jci-135-189511-s007.pdf]

Supplementary Table 1. Summary of Antibodies

Antibody	Manufacturer/Catalog Number	Application	Dilution
RAD51	Abcam/ab133534	Immunofluorescence	1:1000
γH2AX	Millipore-Sigma/05-636	Immunofluorescence	1:500
Phosphorylated RPA2 (Thr21)	ThermoFisher/BS-5693R	Immunofluorescence	1:500
Geminin	Leica, Novacastra/NCL-L Geminin	Immunofluorescence	1:60
Geminin	Proteintech/10802-1-AP	Immunofluorescence	1:400
Alexa Fluor 568, Anti-Ms	Invitrogen, A10037	Immunofluorescence	1:500
Alexa Fluor 568, Anti-Rb	Invitrogen, A11011	Immunofluorescence	1:500
Alexa Fluor 488, Anti-Rb	Invitrogen, A32731	Immunofluorescence	1:500
Alexa Fluor 488, Anti-Ms	Invitrogen, A32723	Immunofluorescence	1:500
Alexa Fluor 647, Anti-Rb	Invitrogen, A32733	Immunofluorescence	1:500
Phosphorylated RPA2 (Thr21)	ThermoFisher/BS-5693R	Western blot	1:1000
HSP70	ThermoFisher/PA5-34772	Western blot	1:5000

A

	Total (n=9)
Age (years)	60.4±12.4
FIGO Stage	
IIIA	1 (11)
IIIB	2 (22)
IIIC	5 (56)
Unknown	1 (11)
Histology	
Serous	7 (78)
Mixed	1 (11)
Unknown	1 (11)
BRCA Mutation	
Yes	1 (11)
No	7 (78)
Unknown	1 (11)
Prior Lines of Chemotherapy	
0	6 (67)
1	2 (22)
Unknown	1 (11)
Platinum Response	
Sensitive (PFI ≥ 6 months)	3 (33)
Resistant (PFI ≤ 6 months)	5 (56)
Unknown	1 (11)

Data are n (%) unless stated otherwise. ± denotes standard deviation.
PFI, Platinum-free interval

B

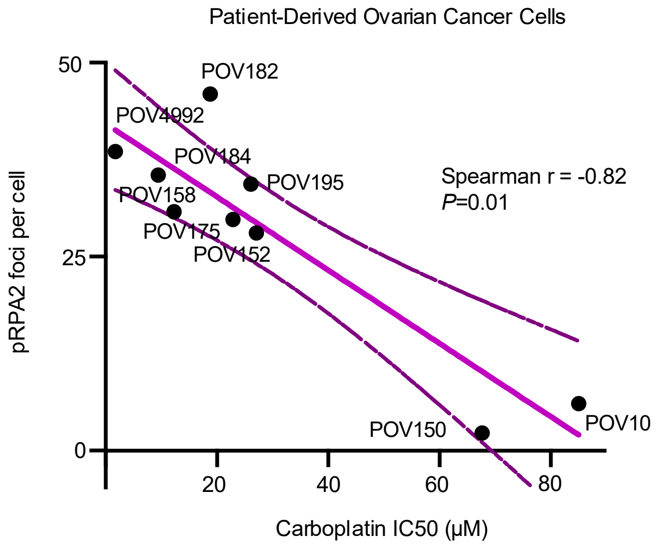

Supplementary Figure 1. pRPA2 foci inversely correlate with platinum chemotherapy resistance in patient-derived ovarian cancer cells (POVs). **A,** Demographic and clinical characteristics of patients from whom primary ovarian cancer cells were collected. **B,** The IC_{50} of carboplatin in 9 POVs was determined and compared to baseline pRPA2 foci of the cells (Spearman r = -0.82, *P*=0.01).

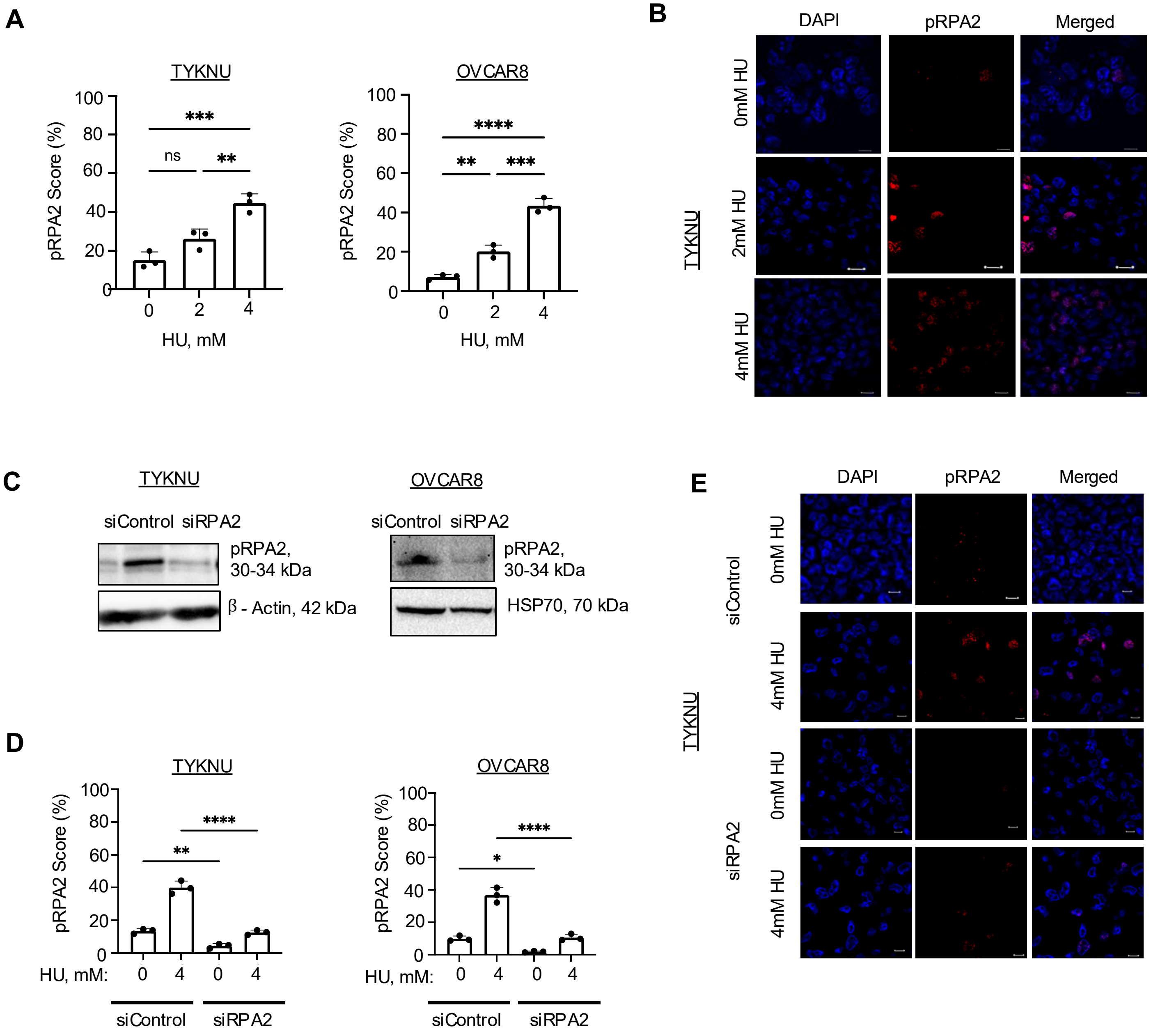

A
TYKNU
pRPA2 Score (%)
HU, mM
ns
OVCAR8
B
DAPI
pRPA2
Merged
0mM HU
2mM HU
4mM HU
TYKNU
C
TYKNU
siControl siRPA2
pRPA2, 30-34 kDa
β - Actin, 42 kDa
OVCAR8
HSP70, 70 kDa
D
HU, mM:
siControl
siRPA2
E
siControl
siRPA2

Supplementary Figure 2. Validation of pRPA2 immunofluorescence assay in formalin-fixed paraffin-embedded (FFPE) samples. A, Dynamic range of pRPA2 score in two FFPE HR-proficient HGSC cell lines after treatment with 0, 2, and 4 mM of hydroxyurea (HU) to induce replication stress. Cells were treated, fixed, embedded, and cut into 4 μm sections for evaluation. **B,** Representative images of DAPI, pRPA2 and colocalization of DAPI/pRPA2 at 63X in an FFPE HGSC cell line treated with varying levels of hydroxyurea. Scale bars: 50 μm. **C,** Western Blot of HGSC cell lines after transfection with siRNA targeting RPA2. **D,** pRPA2 score in two HR-proficient HGSC cell lines after transfection with siRNA targeting RPA2 (siRPA2) or a non-coding region (siControl) and exposed to 0 and 4 mM of hydroxyurea. Cells were treated, fixed, embedded, and cut into 4 μm sections for evaluation. **E,** Representative images of DAPI, pRPA2 and overlay of DAPI/pRPA2 at 63X in FFPE HGSC cells. $*P<0.05$, $**P<0.01$, $***P<0.001$, $****P<0.0001$ by one-way ANOVA.

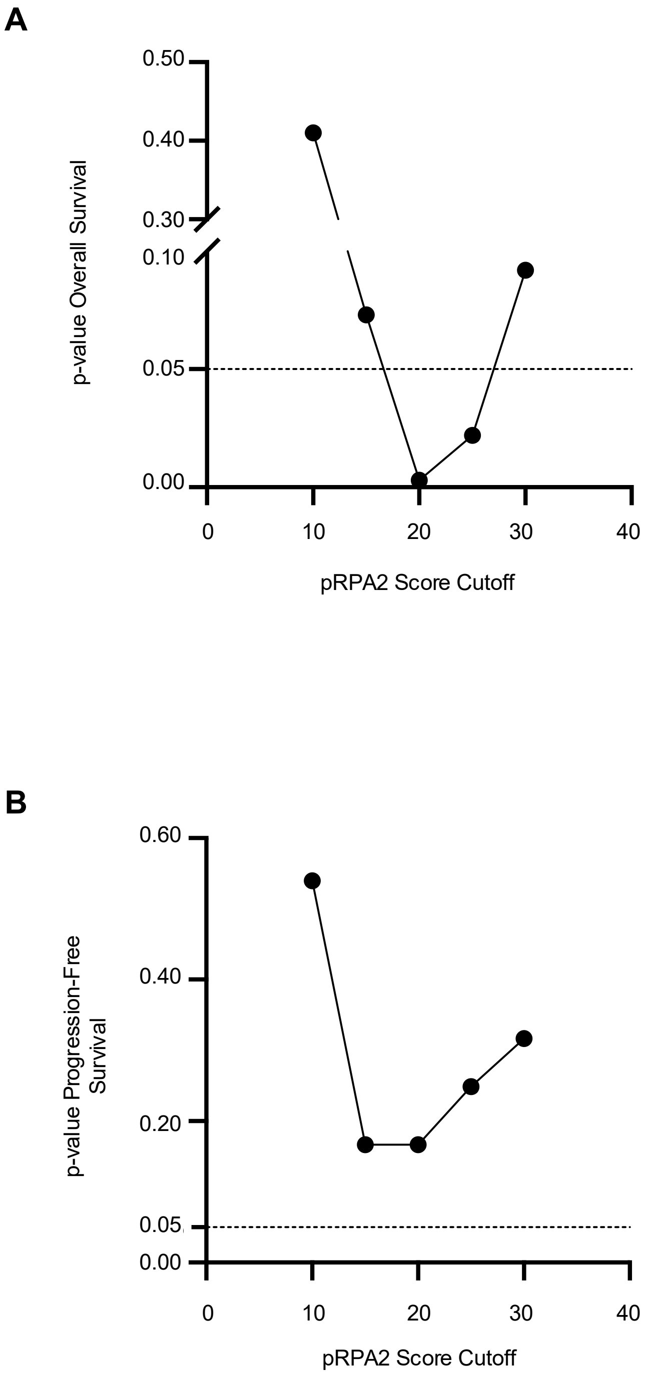
A
p-value Overall Survival
0.50
0.40
0.30
0.10
0.05
0.00
0
10
20
30
40
pRPA2 Score Cutoff
B
p-value Progression-Free Survival
0.60
0.40
0.20
0.05
0.00
0
10
20
30
40
pRPA2 Score Cutoff

Supplementary Figure 3. Optimization of pRPA2 cutoff for survival stratification.

The pRPA2 cutoff (≥20% of cells with ≥2 foci) was determined by optimizing differences in **A,** overall survival and **B,** progression-free survival. Various cutoffs were systematically evaluated, with the final threshold selected for the most significant overall survival and progression-free survival stratification, ensuring robust survival discrimination.

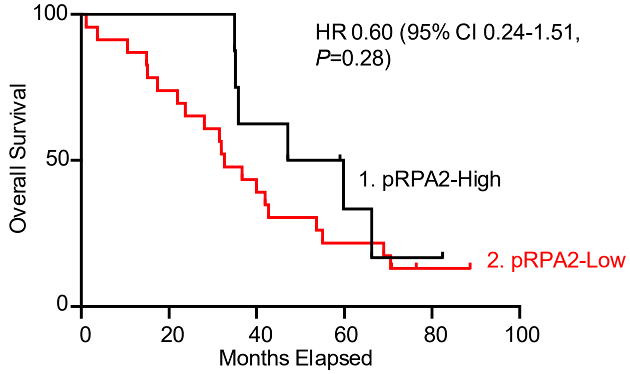

No. at Risk						
1.	8	8	5	2	1	0
2.	23	17	9	5	1	0

Supplementary Figure 4. pRPA2 score alone does not predict overall survival in a discovery cohort. Kaplan-Meier curves evaluating overall survival in patients with HGSCs stratified by pRPA2 score (n=31, HR 0.6 95% CI 0.2-1.5, *P*=0.3).

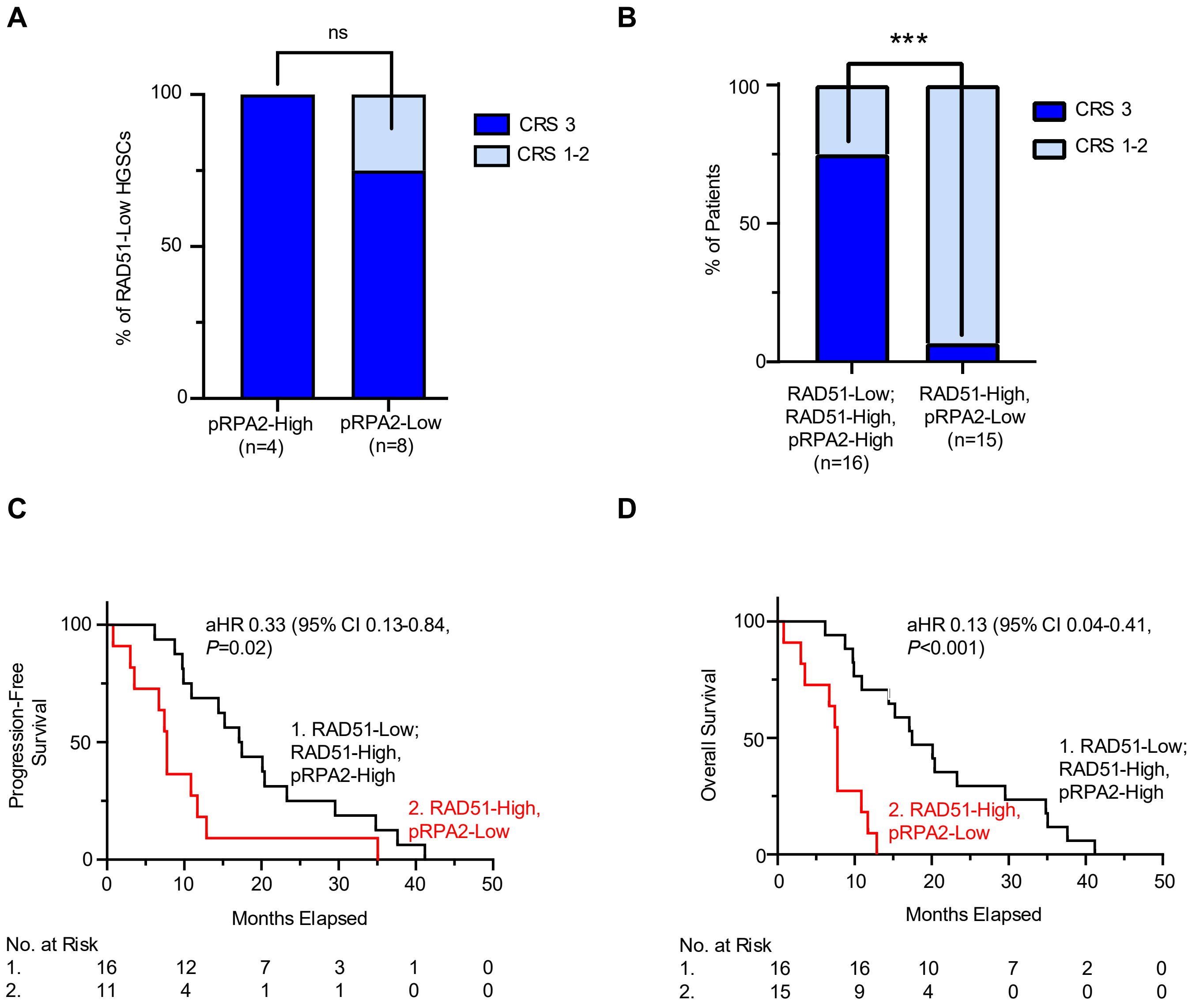
A
ns
100
50
0
% of RAD51-Low HGSCs
pRPA2-High
(n=4)
pRPA2-Low
(n=8)
CRS 3
CRS 1-2
B
***
100
50
0
% of Patients
RAD51-Low;
RAD51-High,
pRPA2-High
(n=16)
RAD51-High,
pRPA2-Low
(n=15)
CRS 3
CRS 1-2
C
100
50
0
Progression-Free
Survival
aHR 0.33 (95% CI 0.13-0.84,
P=0.02)
1. RAD51-Low;
RAD51-High,
pRPA2-High
2. RAD51-High,
pRPA2-Low
0
10
20
30
40
50
Months Elapsed
No. at Risk
1. 16 12 7 3 1 0
2. 11 4 1 1 0 0
D
100
50
0
Overall Survival
aHR 0.13 (95% CI 0.04-0.41,
P<0.001)
1. RAD51-Low;
RAD51-High,
pRPA2-High
2. RAD51-High,
pRPA2-Low
0
10
20
30
40
50
Months Elapsed
No. at Risk
1. 16 16 10 7 2 0
2. 15 9 4 0 0 0

Supplementary Figure 5. RAD51-Low and RAD51-High, pRPA2-High HGSCs are more sensitive to platinum chemotherapy than RAD51-High, pRPA2-Low HGSCs in a discovery cohort. A, Proportion of patients with chemotherapy response score (CRS) of 3 vs CRS of 1-2 in RAD51-Low HGSCs with either pRPA2-High or pRPA2-Low score P=0.52. **B,** CRS in RAD51-Low or RAD51-High, pRPA2-High HGSCs vs RAD51-High, pRPA2-Low HGSCs (Relative Risk 11.3, 95% CI 1.7-76.3, P<0.001). Kaplan-Meier curves evaluating **C,** progression-free survival (n=27, aHR 0.33, 95% CI 0.13-0.84, P<0.001) and **D,** overall survival (n=31, aHR 0.13, 95% CI 0.04-0.41, P<0.001) in patients with HGSCs stratified by RAD51 and pRPA2 scores. ***P<0.001 by student's two-tailed t-test. aHR= adjusted hazard ratio for: age, stage, residual disease and *BRCA* status.

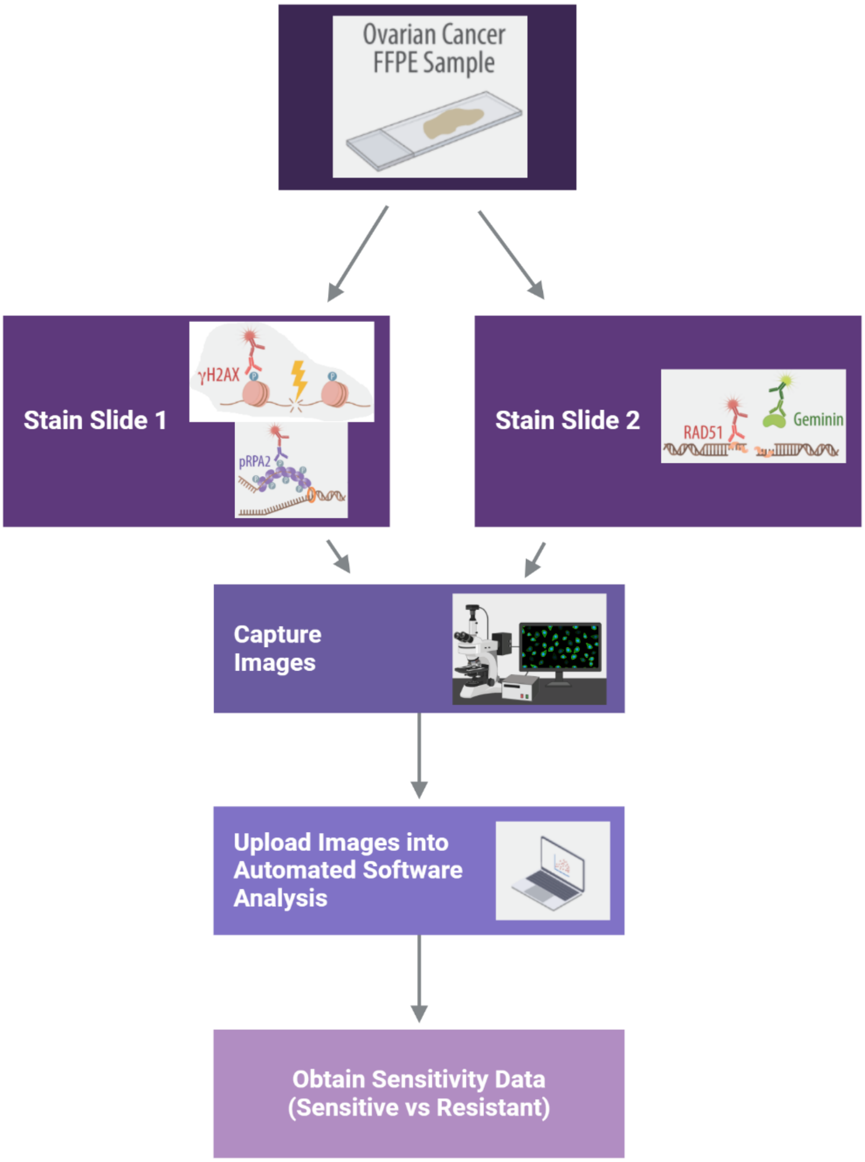
Ovarian Cancer FFPE Sample
Stain Slide 1
γH2AX
pRPA2
Stain Slide 2
RAD51
Geminin
Capture Images
Upload Images into Automated Software Analysis
Obtain Sensitivity Data (Sensitive vs Resistant)

Supplementary Figure 6. Schematic of functional homologous recombination and replication stress assay. Two FFPE slides are used, one is stained for pRPA2 and γH2AX and the other is stained for RAD51 and geminin. After imaging, images are uploaded to automated quantification software to obtain a score which is used to predict sensitivity or resistance.

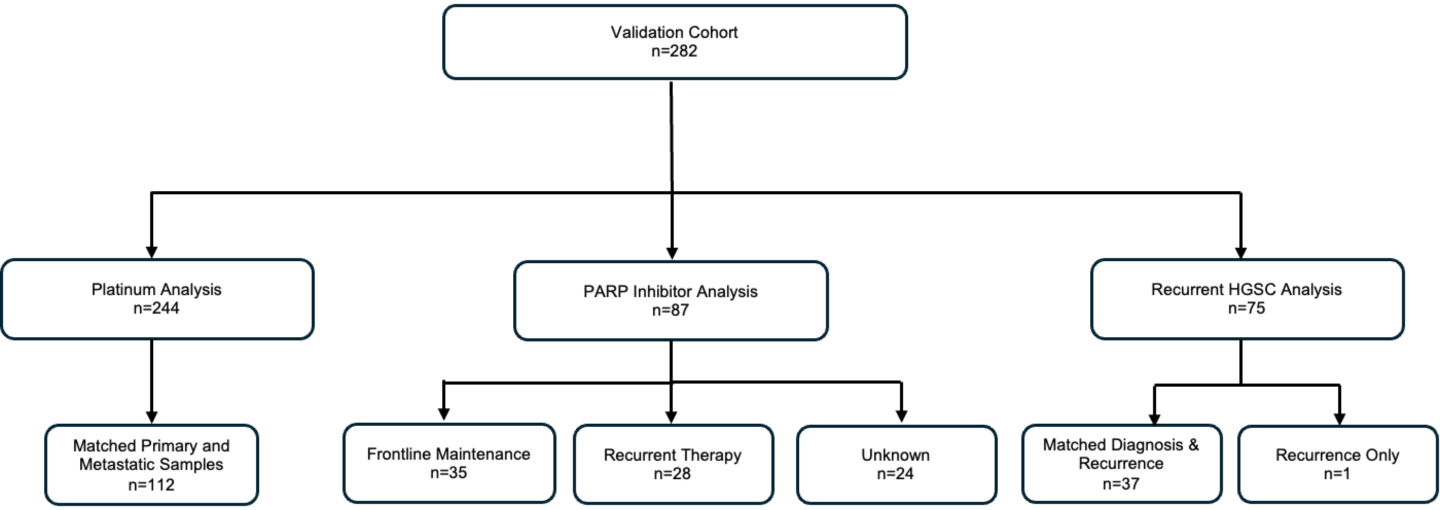
Validation Cohort
n=282
Platinum Analysis
n=244
PARP Inhibitor Analysis
n=87
Recurrent HGSC Analysis
n=75
Matched Primary and
Metastatic Samples
n=112
Frontline Maintenance
n=35
Recurrent Therapy
n=28
Unknown
n=24
Matched Diagnosis &
Recurrence
n=37
Recurrence Only
n=1

Supplementary Figure 7. Flow diagram of patient-derived HGSC samples included in analyses.

A

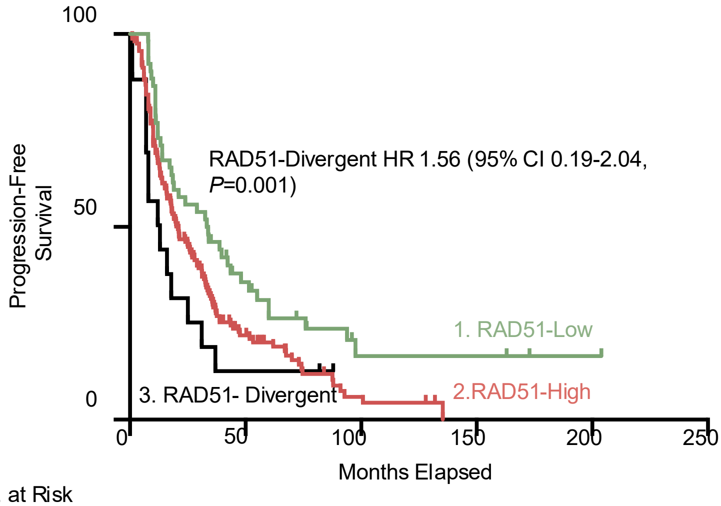

No. at Risk						
1.	52	16	4	4	2	0
2.	165	25	4	0	0	0
3.	19	2	0	0	0	0

B

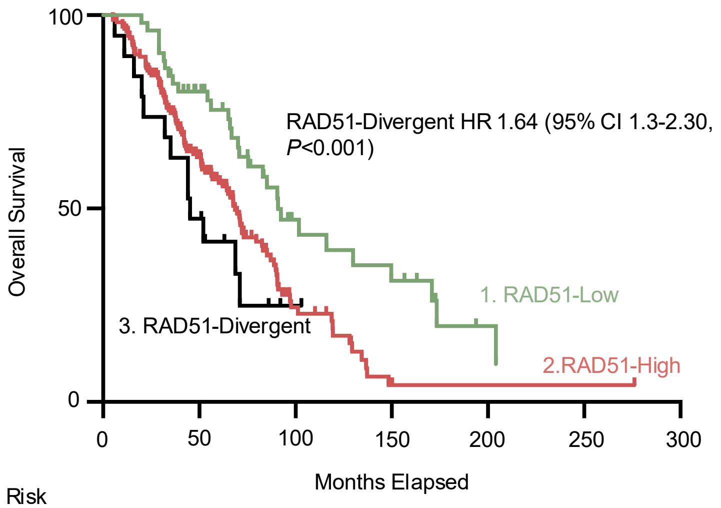

No. at Risk							
1.	51	36	12	8	2	0	0
2.	170	81	15	2	1	1	0
3.	19	9	1	0	0	0	0

Supplementary Figure 8. Intra-tumoral RAD51 heterogeneity predicts decreased survival in patients with HGSCs treated with platinum chemotherapy. Kaplan-Meier curves evaluating **A,** progression-free survival (n=236, RAD51-Divergent HR 1.56, 95% CI 0.19-2.04, *P*=0.001) and **B,** overall survival in patients with RAD51-High, RAD51-Low, or RAD51-Divergent HGSCs (n=240, RAD51-Divergent HR 1.64, 95% CI 1.3-2.30, *P*<0.001).

A

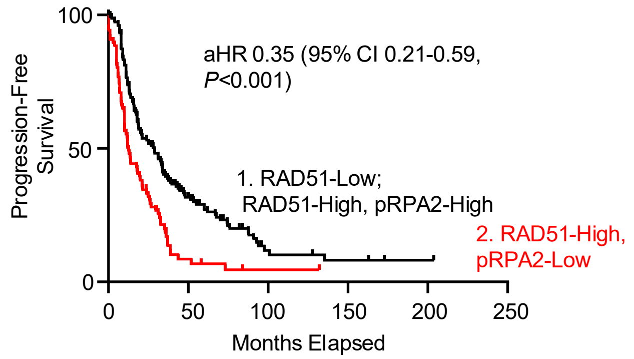

No. at Risk

1.	151	26	5	4	2	0
2.	87	5	1	0	0	0

B

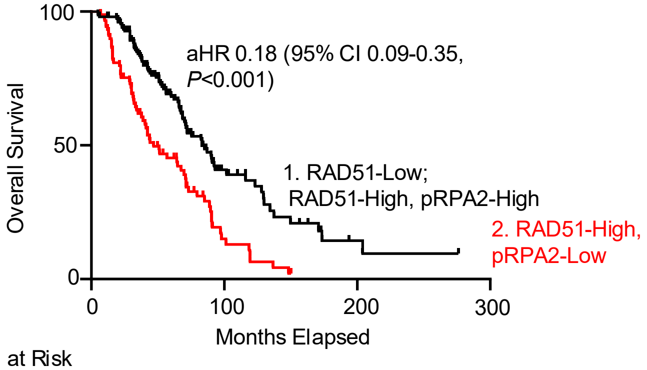

No. at Risk

1.	154	22	3	0
2.	89	7	0	0

Supplementary Figure 9. RAD51-Low and RAD51-High, pRPA2-High HGSCs are more sensitive to platinum chemotherapy than RAD51-High, pRPA2-Low HGSCs in a validation cohort. Kaplan-Meier curves evaluating **A,** progression-free survival (n=238, aHR 0.35, 95% CI 0.21-0.59 $P<0.001$) and **B,** overall survival (n=243, aHR 0.18, 95% CI 0.09-0.35 $P<0.001$) in patients with HGSCs stratified by RAD51 and pRPA2 scores. aHR= adjusted hazard ratio for: age, stage, residual disease and *BRCA* status.

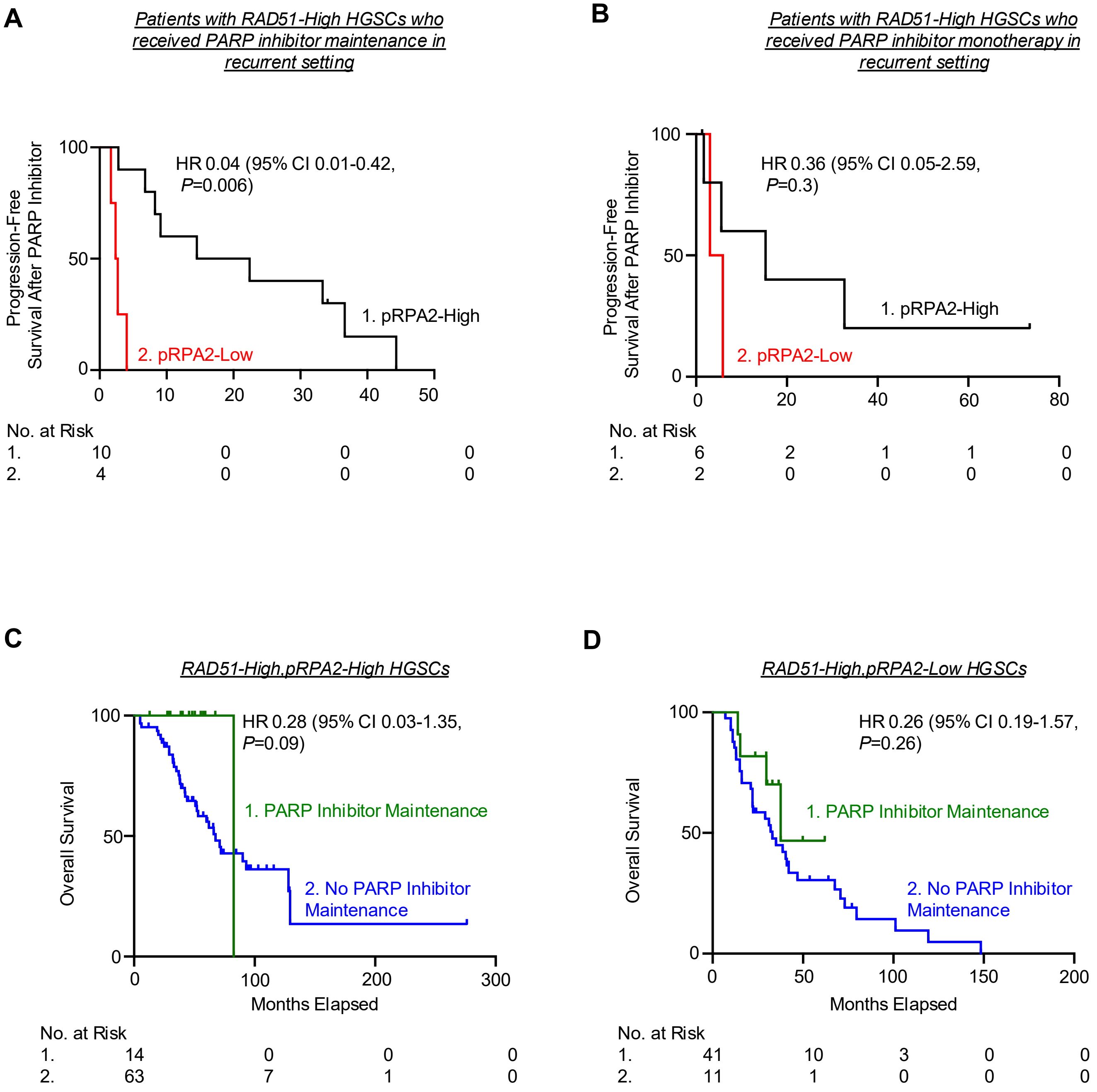
A
Patients with RAD51-High HGSCs who received PARP inhibitor maintenance in recurrent setting
Progression-Free Survival After PARP Inhibitor
HR 0.04 (95% CI 0.01-0.42, P=0.006)
1. pRPA2-High
2. pRPA2-Low
0 10 20 30 40 50
100 50 0
No. at Risk
1. 10 0 0 0
2. 4 0 0 0
B
Patients with RAD51-High HGSCs who received PARP inhibitor monotherapy in recurrent setting
Progression-Free Survival After PARP Inhibitor
HR 0.36 (95% CI 0.05-2.59, P=0.3)
1. pRPA2-High
2. pRPA2-Low
0 20 40 60 80
100 50 0
No. at Risk
1. 6 2 1 1 0
2. 2 0 0 0 0
C
RAD51-High,pRPA2-High HGSCs
Overall Survival
HR 0.28 (95% CI 0.03-1.35, P=0.09)
1. PARP Inhibitor Maintenance
2. No PARP Inhibitor Maintenance
Months Elapsed
0 100 200 300
100 50 0
No. at Risk
1. 14 0 0 0
2. 63 7 1 0
D
RAD51-High,pRPA2-Low HGSCs
Overall Survival
HR 0.26 (95% CI 0.19-1.57, P=0.26)
1. PARP Inhibitor Maintenance
2. No PARP Inhibitor Maintenance
Months Elapsed
0 50 100 150 200
100 50 0
No. at Risk
1. 41 10 3 0 0
2. 11 1 0 0 0

Supplementary Figure 10. Automated pRPA2 score predicts survival in patients with RAD51-High HGSCs treated with PARP inhibitors. A, Kaplan-Meier curves evaluating progression-free survival in patients with RAD51-High HGSCs who received PARP inhibitor maintenance in the recurrent setting stratified by pRPA2 score (n=14, HR 0.04, 95% CI 0.01-0.42, *P*=0.006). **B,** Kaplan-Meier curves evaluating progression-free survival in patients with RAD51-High HGSCs who received PARP inhibitor monotherapy in the recurrent setting stratified by pRPA2 score (n=8, HR 0.36, 95% CI 0.05-2.59, *P*=0.3). **C,** Kaplan-Meier curves evaluating overall survival in patients with RAD51-High, pRPA2-High HGSCs treated with or without frontline PARP inhibitor maintenance therapy (n=77, HR 0.28, 95% CI 0.03-1.35, *P*=0.09). **D,** Kaplan-Meier curves evaluating overall survival in patients with RAD51-High, pRPA2-Low HGSCs treated with or without frontline PARP inhibitor maintenance therapy (n=52, HR 0.26, 95% CI 0.19-1.57, *P*=0.26).

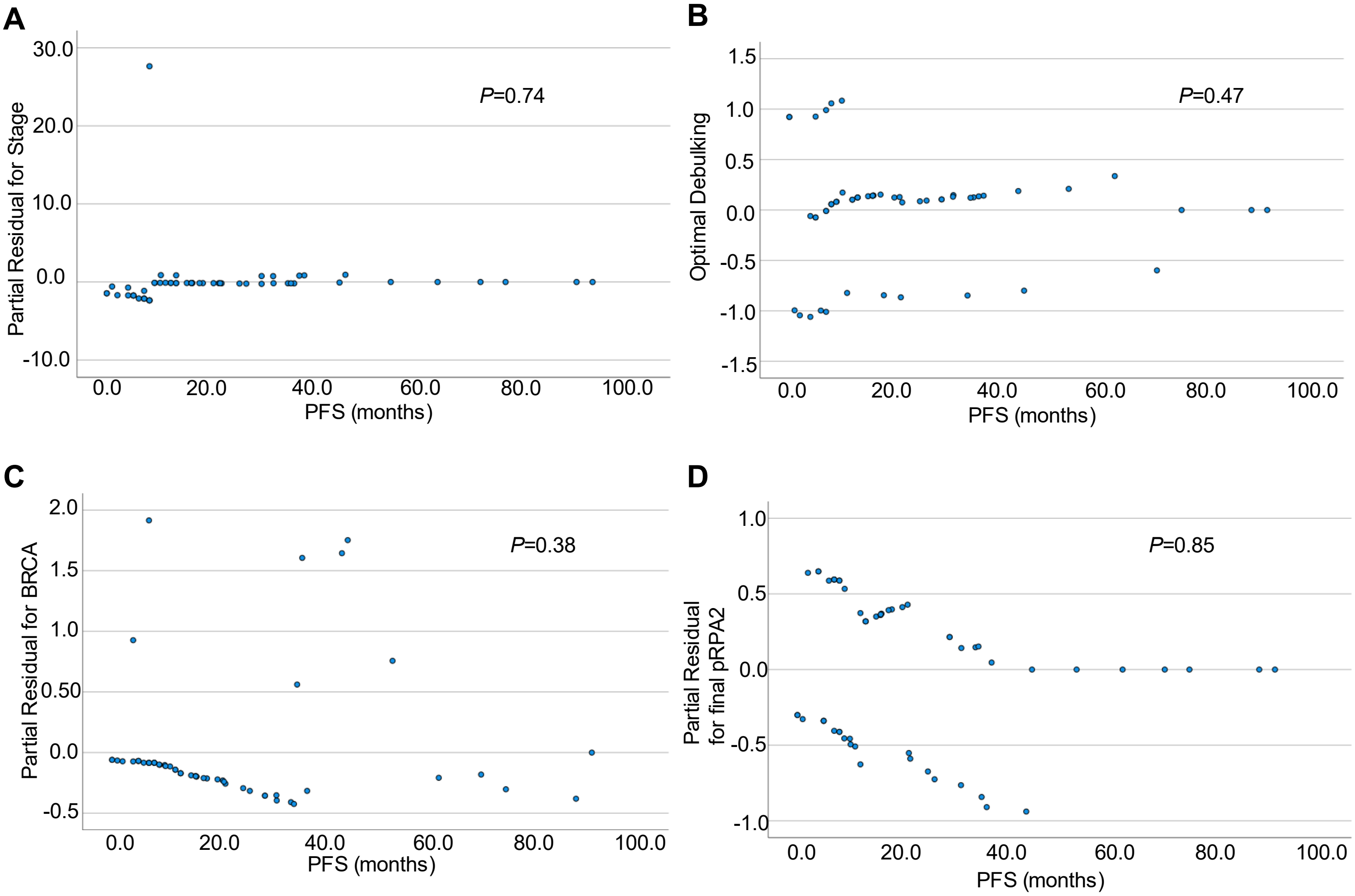
A
Partial Residual for Stage
P=0.74
PFS (months)
B
Optimal Debulking
P=0.47
PFS (months)
C
Partial Residual for BRCA
P=0.38
PFS (months)
D
Partial Residual for final pRPA2
P=0.85
PFS (months)

Supplementary Figure 11. Assessment of the proportional hazards assumption using Schoenfeld residuals. A-D, Schoenfeld residuals plotted against time for covariates included in the Cox proportional hazards model. The residuals should exhibit no clear trend over time and therefore the proportional hazards assumption holds.
