# Supplementary material for: Phospho-RPA2 predicts response to platinum and PARP inhibitors in homologous recombination–proficient ovarian cancer
Source: J Clin Invest. 2025 May 20;135(13):e189511. doi: 10.1172/JCI189511 (PMC12208538; doi:10.1172/JCI189511)
Supplement: Unedited blot and gel images [file jci-135-189511-s009.pdf]

Uncropped blots for Supplemental Figure 2C
Bands in red box corresponds to what is shown in the Supplemental Figure

TYKNU

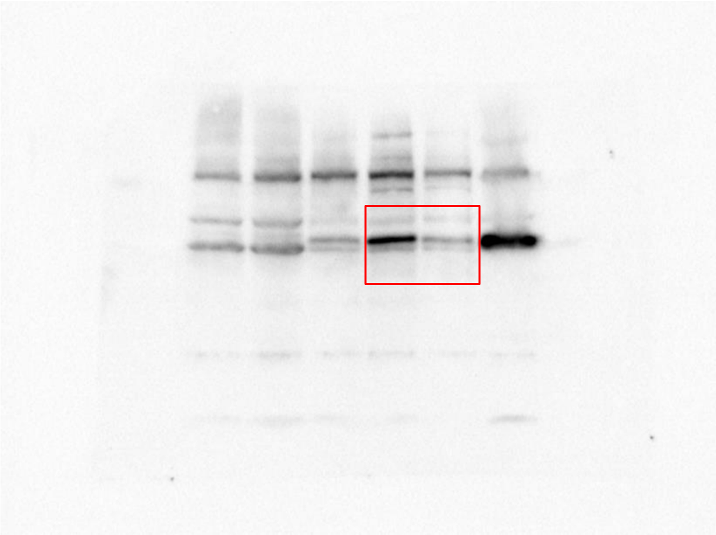

pRPA2

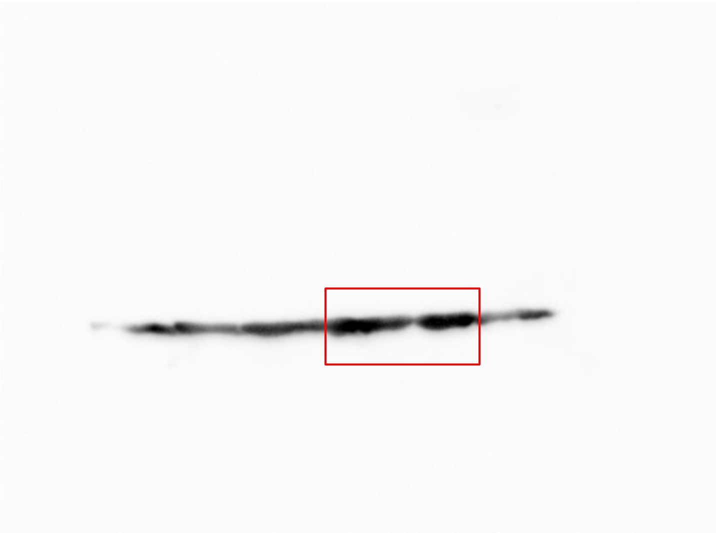

β - Actin 42 kDa

OVCAR8

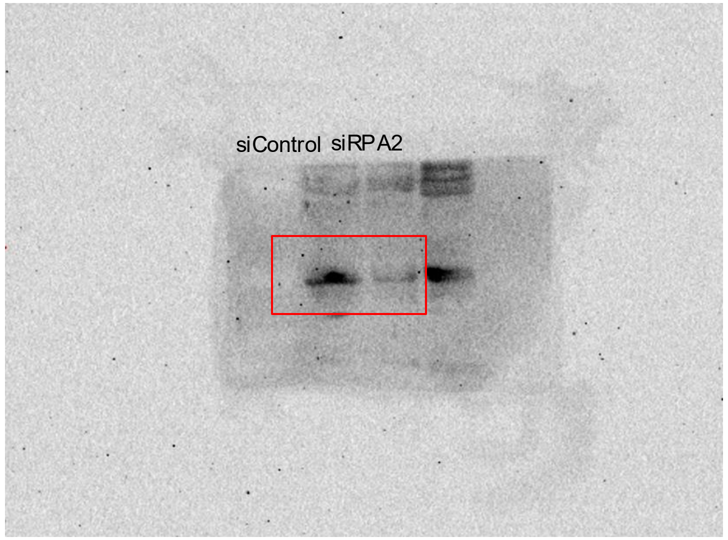

pRPA2

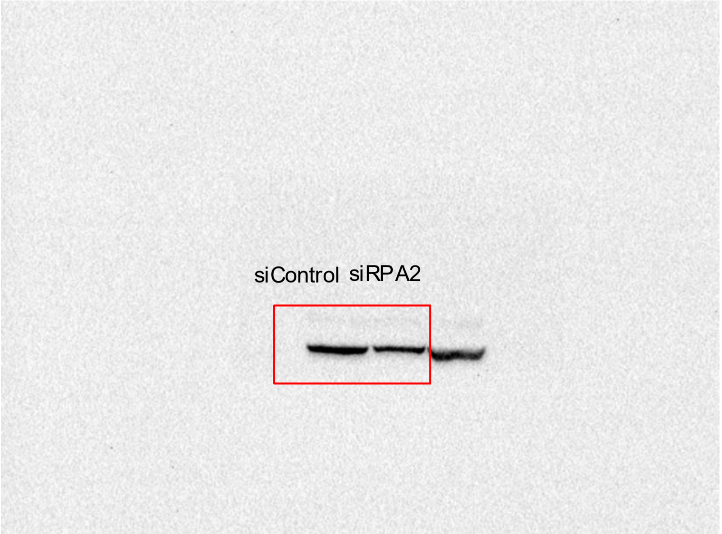

HSP70
